# Red-Shifted Aminated Derivatives of GFP Chromophore for Live-Cell Protein Labeling with Lipocalins

**DOI:** 10.3390/ijms19123778

**Published:** 2018-11-28

**Authors:** Nina G. Bozhanova, Mikhail S. Baranov, Nadezhda S. Baleeva, Alexey S. Gavrikov, Alexander S. Mishin

**Affiliations:** 1 Institute of Bioorganic Chemistry, Russian Academy of Sciences, Miklukho-Maklaya 16/10, 117997 Moscow, Russia; nbozhanova@gmail.com (N.G.B.); dyuha-89@yandex.ru (N.S.B.); gavrikovalexey1@gmail.com (A.S.G.); 2 Pirogov Russian National Research Medical University, Ostrovitianov 1, 117997 Moscow, Russia

**Keywords:** fluorogenic dyes, arylmethane dyes, GFP chromophore, fluorescent labeling, fluorescence microscopy, protein-PAINT

## Abstract

Fluorogens are an attractive type of dye for imaging applications, eliminating time-consuming washout steps from staining protocols. With just a handful of reported fluorogen-protein pairs, mostly in the green region of spectra, there is a need for the expansion of their spectral range. Still, the origins of solvatochromic and fluorogenic properties of the chromophores suitable for live-cell imaging are poorly understood. Here we report on the synthesis and labeling applications of novel red-shifted fluorogenic cell-permeable green fluorescent protein (GFP) chromophore analogs.

## 1. Introduction

Fluorogenic dyes are compounds which are non-fluorescent by themselves in solution but become highly emissive in a complex with the target molecule. The list of target molecules compatible with cellular imaging applications includes RNA aptamers [1], and various proteins [2,3,4,5]. These fluorogen–target pairs hold the promise for simple no-washout cellular staining, an increase in the signal-to-noise ratio, and photostability in cellular imaging applications. 

Rational design of novel bipartite tags (fluorogen–target pairs) requires understanding the molecular mechanisms behind the fluorogenic behavior. There are two major ways of altering the balance of radiative and non-radiative processes: Conformational lock and exploitation of environmentally sensitive non-fluorescent excited state, for instance, the state with internal charge transfer (ICT). 

Various arylmethane compounds typically have no fluorescence in the free state in solution due to their intrinsic structural flexibility [6] while the internally-fixed analogs of the same compounds, such as the well-known xanthene dyes with an oxygen fixing bridge [7] or the dyes from BODIPY (boron-dipyrromethene) family with difluoroboric fixing bridge [8], are typically highly-fluorescent [9]. Fixation can also be induced by external influence, therefore, some of di- and triarylmethane dyes have been already used as fluorogens for imaging: With single-chain antibodies [2] or other proteins-based designed binders [3,5]. The chromophores of GFP-like fluorescent proteins are diarylmethane dyes, too, and they also are highly fluorogenic [10]. Cell-permeability of these compounds makes them highly attractive for live-cell imaging of specific RNA [1,11] and protein molecules [3,4]. 

On the other hand, aminated analogs of GFP chromophore demonstrate a high degree of solvatochromism and environment-dependent fluorescence quantum yield (FQY) variation. Presence of the amine group leads to the formation of an additional non-fluorescent excited state with a high degree of charge separation (ICT state) [12,13]. The formation of the ICT state and its stability depend on the environment [14] and can be hindered by the fluorogen-activating protein host. 

**Scheme 1:**
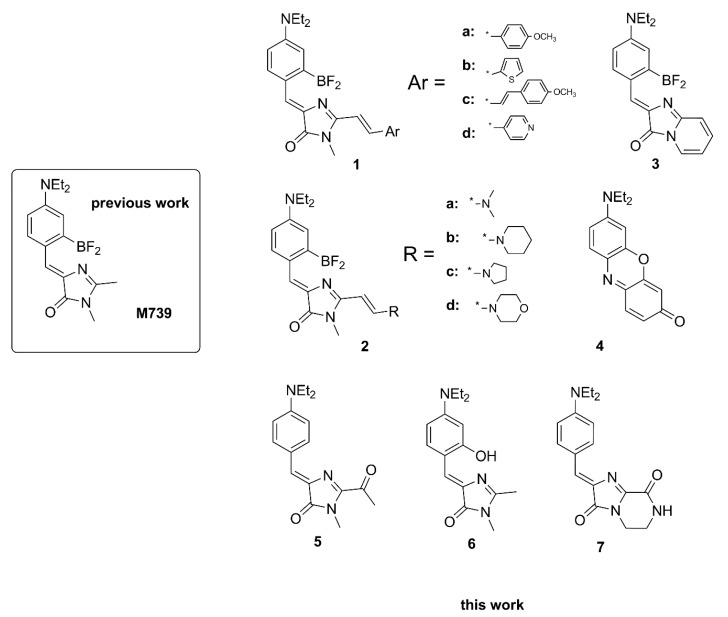
Various analogs of compound **M739**.

In our previous work, we developed protein hosts capable of recovering the fluorescence of such chromophores (primarily compound **M739** [4], Scheme 1), and demonstrated that restriction of flexibility is not the only way to induce fluorogenic behavior.

In this work, we explore a panel of **M739** derivatives (Scheme 1), aiming to create protein–dye bipartite tags with red-shifted fluorescence.

## 2. Results and Discussion

In order to achieve the bathochromic shift in spectra, we followed the conventional approaches: The increases of the length of the conjugated pi-system and the introduction of electron-withdrawing or electron-donating heteroatomic groups. Indeed, GFP-like chromophores of natural red fluorescent proteins are just the extension of the benzylidene-imidazolones (BDI) scaffold with a ketone (AsFP), acylimine (dsRed), or styrene (Kaede) group [15]. Similar modifications of the synthetic GFP chromophores’ analogs were used for the creation of red-shifted fluorogenic dyes for RNA aptamers [11,16]. 

According to the two above mentioned mechanisms, we have created two groups of chromophores: Conformationally locked by fluoroboric or oxygen bridge compounds **1**–**4,** and non-locked compounds **5**–**7** (Scheme 1). 

**Scheme 2:**
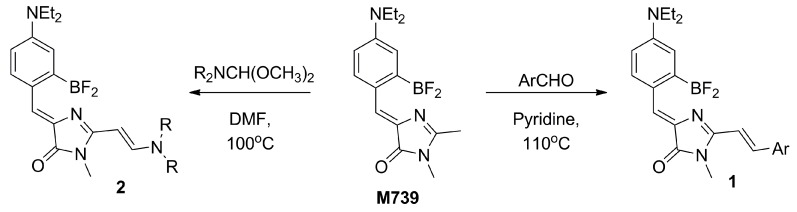
Synthesis of **1** and **2**: analogs of compound **M739** and Kaede chromophore.

**Scheme 3:**
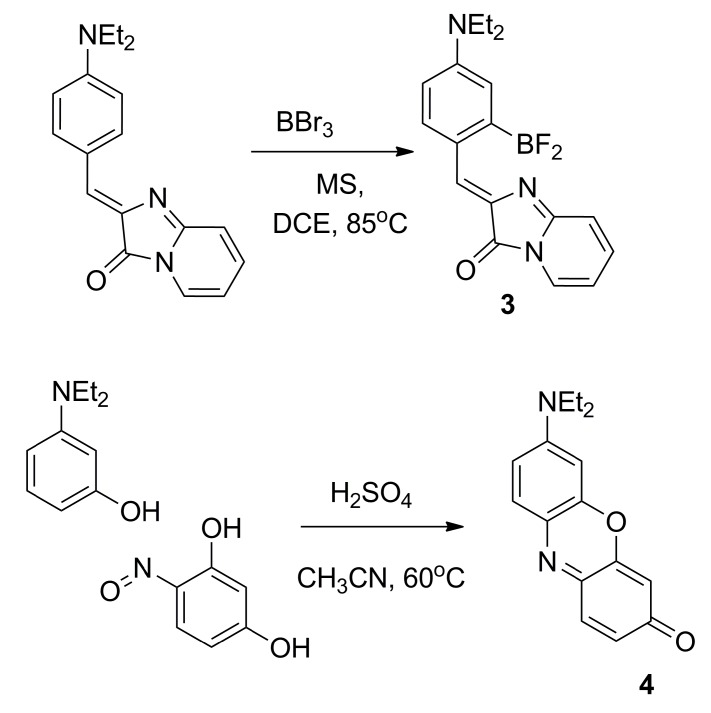
Synthesis of compounds **3** and **4**: locked analogs of compound **M739**.

Compounds **1** and **2** containing an additional free double bond were synthesized by condensation of the borylated compound **M739** with a range of aromatic aldehydes or formamide acetals (Scheme 2) [17]. Locked compound **3** was synthesized as we reported previously by direct borylation of corresponding non-borylated derivative (Scheme 3) [17]. The oxygen bridge containing locked chromophore **4** was created using the condensation between 3-(diethylamino)phenol and nitroso resorcinol (Scheme 3). 

Compound **4** [18] doesn’t belong the GFP chromophore family, but it is geometrically close to compound **M739** and also resembles the structure of Nile-Red dye which is a very promising fluorogenic agent with a noticeable degree of solvatochromism [19].

**Scheme 4:**
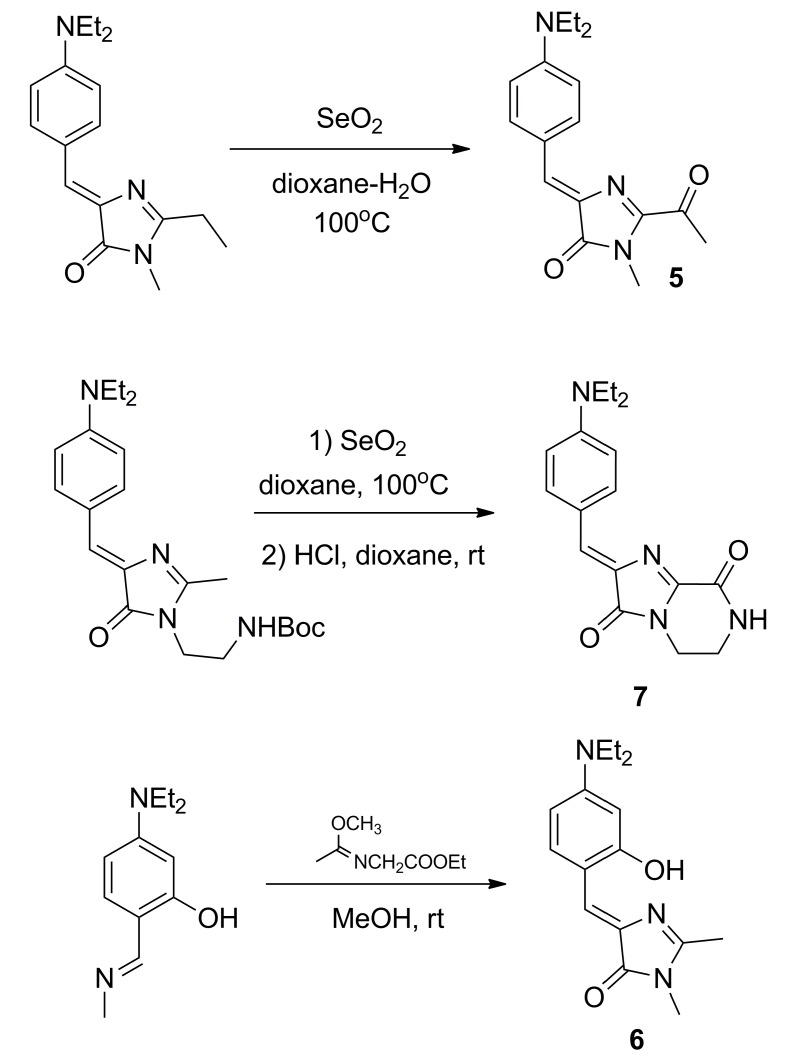
Synthesis of compounds **5**–**7**: Non-locked analogs of compound **M739**.

Selenium dioxide oxidation was used in the synthesis of the non-locked compounds **5** and **7**. This oxidation is a typical approach in the synthesis of red-shifted derivatives of the GFP chromophore [20,21] The compound **6** is similar to the **M739**, with the difluoroboryl group, potentially responsible for the protein binding, replaced with the hydroxyl group. The synthesis of compound **6** was carried out according to a standard procedure using carboxymidate (Scheme 4).

**Figure 1:**
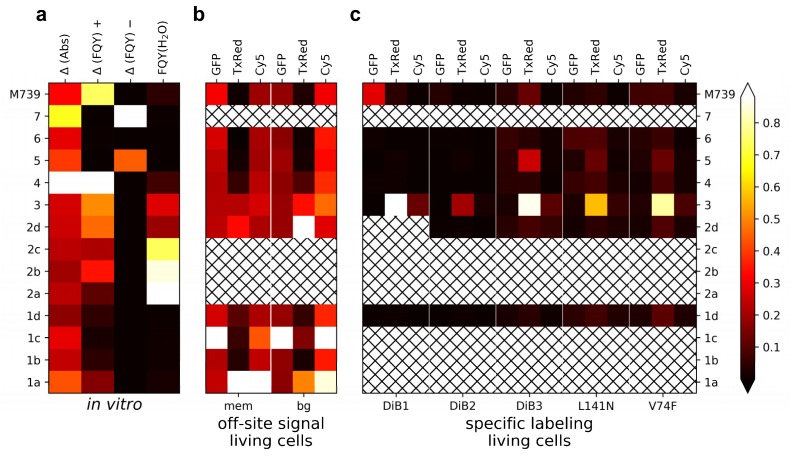
Fluorogenic compounds screening pipeline. The values are normalized column-wise and depicted in pseudocolor. Regions with no data are hatched. (**a**) In vitro screening (see also ESI Table S1): Δ(Abs)—maximum change in absorbance maxima position, relative to water; Δ(FQY)+—maximum increase in fluorescence quantum yield, relative to water; Δ(FQY)—maximum decrease in fluorescence quantum yield, relative to water; FQY(H_2_O)—fluorescence quantum yield in water. (**b**) Off-site signal and background in HeLa cells: Mem-signal in membranes, bg—signal in the imaging media. (**c**) Specific labeling in HEK293 cells expressing H2B-fusions of lipocalin mutants.

We conducted a stepwise screening in order to find compounds suitable for live-cell imaging (Figure 1). First, we determined the optical properties of the created substances. The absorbance, fluorescence, and solvatochromic properties of the synthesized compounds were notably altered in comparison with the **M739** (ESI Table S1). The introduction of the electron-withdrawing groups and extended conjugated pi-systems into the imidazolone moiety were previously found to be the most effective approaches to achieve the red-shift of the BDIs’ spectra [17]. In compliance with earlier observations, compounds **1** containing both modifications indeed exhibited the most prominent bathochromic shifts, reaching 80-94 nm for absorption and up to 165 nm for emission. 

In contrast, the spectra of enamine derivatives **2** were shifted by no more than 10–30 nm. These compounds also contain an additional pi-system, but the substituent in it is electron-donating one. Introduction of long pi-system without any additional groups in compound **3** leads to the red-shift up to 90 nm. The maximum bathochromic shift achieved in oxidized derivatives **5** and **7** was only 10 nm (in some solvents, they showed a hypsochromic shift of the same magnitude). These compounds contain only small electron-withdrawing groups. The ‘mimicking’ compound **4**, in which imidazolone ring is modified, was also highly red-shifted (50–90 nm). This modification cannot be defined clearly in terms mentioned above, but nitrogen bridge and carbonyl group are electron-withdrawing.

The other way to achieve the red-shift of the BDIs’ spectra is the introduction of electron-donating groups into the benzylidene moiety [12]. From the previously obtained data [4] we know that addition of bulky substituents into this moiety leads to the dramatic increase of *K*_d_. Thus, in present work we introduce only a small OH group in place of the BF2 in the M739 core, resulting in compound **6**. However, this modification was found to be insufficient to achieve the pronounced redshift, but it possibly can improve the chromophore-protein binding (due to the presence of polar and H-binding group in the same place) or signal-back noise ratio (compound **6** has low FQY in free state in all solvents).

Not all of the observed solvent-dependent changes in FQY were beneficial for potential imaging applications. Even though most of the compounds showed the high degree of FQY variation between solvents (ESI Table S1) with the tendency of FQY decrease in polar and protic environment (i.e., water), the majority of compounds demonstrated high FQY in all solvents. That would result in high background fluorescence in water-based imaging media utilized in live-cell fluorescence microscopy.

Therefore, after the in vitro stage of screening of the compounds (Figure 1a), we excluded from consideration compounds **2a**, **2b**, and **2c** due to the high FQY values in water (ESI Table S1, Figure 1a), and compound **7** due to the lack of any signs of solvatochromic or fluorogenic properties.

On the second step of screening, we investigated the pH-dependence of spectra of the chromophores because it may alter the labeling efficiency, as it was reported previously for other aminated derivatives of GFP chromophore [12]. Acidification of the solutions of all compounds **1**–**7** led to a noticeable hypsochromic shift in the absorption spectra caused by the protonation of the aromatic amino group. However, the p*K*_a_ of this process was less than 4 in all cases, making all of the synthesized compounds’ spectra pH-independent in physiological conditions.

On the third step (Figure 1b), we proceeded with screening for background and non-specific fluorescence on the model of cultured mammalian cells. The composition of imaging media for prolonged live-cell microscopy includes proteins of the albumin family, which may bind GFP-like chromophores and alter their fluorescence [22]. Also, we assessed the fluorescence in cellular membranes. The compounds with a strong fluorescence in the imaging media, cellular membranes or cytoplasm were excluded from further tests. Finally, we tested compounds **1d**, **2d**, and **3**–**6** for the ability to specifically bind fluorogen-activating proteins in cells. 

In the last stage of screening, we assessed the magnitude and similarity of the signals from the reference channel (blue fluorescent protein TagBFP) and the target signal in the green-red channels (Figure 1, ESI) in living transiently transfected cells, expressing chimeric proteins of histone H2B, fluorescent protein TagBFP, and lipocalin mutants Blc-L141N, Blc-V74F, and DiBs 1–3 [4] (Blc-A36C/L141N (DiB1), Blc-A36C (DiB2), Blc-V74F/L141Q (DiB3)). The images were acquired 10 minutes after the addition of the compounds (final concentration 0.1–1 µM) to the media. Therefore, the dyes with slow cell-penetration kinetics were filtered out. 

Thus, we selected the only candidate—compound **3**. This compound exhibited the most robust fluorescence increase in the red detection channel among all the chromophores tested in cellulo. In vitro titration showed submicromolar *K*_d_s for most of the mutants (Table 1). The best-performing complex with lipocalin DiB1 exhibited 90 nM *K*_d_ and the FQY of 0.29. 

**Figure 2:**
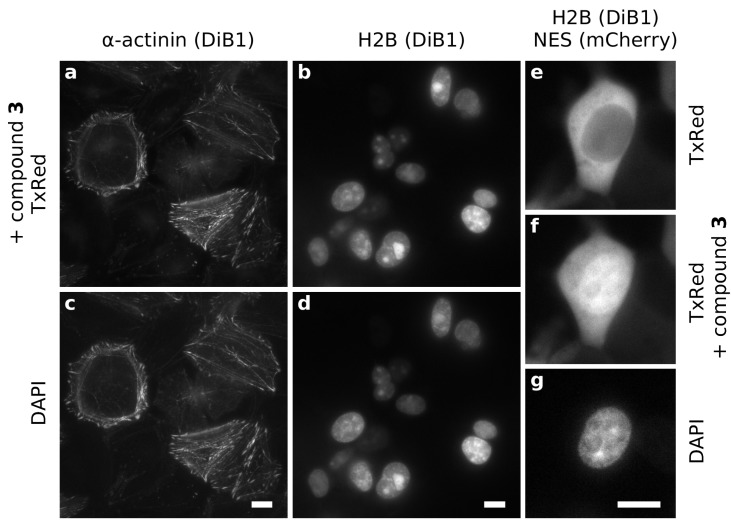
Live-cell protein labeling using DiB1: Compound **3** pair. (**a**,**b**,**e**,**f**) target channel (DiB1 imaged with TxRed filter set in the presence of 100 nM of the compound **3**), (**c**,**d**,**g**) reference channel (TagBFP imaged with DAPI filter set), (**a**,**c**) alpha-actinin, HeLa cells (**b**,**d**) histone H2B, HEK293 cells (**e**–**g**) HEK293 cells transiently co-transfected with mCherry-NES (nuclear export signal) and TagBFP-DiB1-H2B, before (**e**) and 5 min after (**f**) addition of the compound **3** (100 nM). Scale bars 10 μm.

Despite modest fluorescence increase in vitro (~2.3 times) with respect to the water, we achieved efficient labeling of target proteins (histone H2B, alpha-actinin) with DiB1 in living cells by incubating the cells with 100 nM of the compound **3** (Figure 2). Histone H2B labeled with DiB1: Compound **3** complex (FQY 0.29) was distinguishable in cells transiently expressing red fluorescent protein mCherry fused with a nuclear export signal (Figure 2e,f). We found the photostability of DiB1: Compound **3** complex to be similar to the one of spectrally similar red fluorescent protein mKate2 (ESI Figure S1).

**Figure 3:**
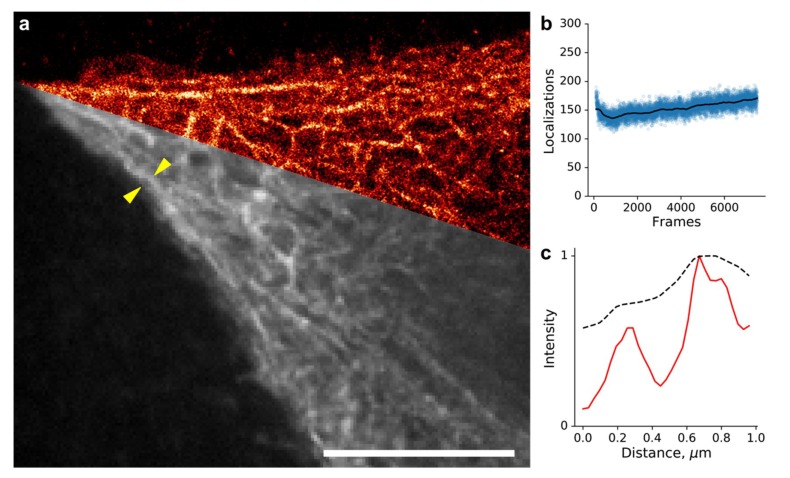
Super-resolution imaging with the DiB1 in the presence of compound **3**. (**a**) Live HeLa Kyoto cells were transiently transfected with cytokeratin-18-DiB1 and imaged in the presence of compound **3** (15 nM). Overlay of widefield (bottom) image and super-resolution reconstruction (top, from 3000 frames) are shown; scale bars are 10 µm. (**b**) The graph shows the number of localizations per frame. The laser illumination occurred without intermittence, and the frames were taken with 16 ms exposure. (**c**) Normalized intensity profiles between the arrowheads shown on the widefield image; black curve—widefield and red curve—super-resolution. The fluorescence was excited with 561 nm (60 W/cm^2^) laser line.

Similarly, to the previously published lipocalin-fluorogen pairs [4], the reversible complex of DiB1 and compound **3** can be used as a bipartite protein-PAINT tag for super-resolution of proteins of interest in living cell. We tested performance of the DiB1: Compound **3** complex in a single-molecule super-resolution setup. Upon addition of 15 nM of compound **3** to imaging media we observed constant number of single-molecule fluorescence bursts for more than 20 min of uninterrupted image acquisition. Light-independent control of the number of localization events per frame allow for super-resolution image reconstruction with illumination power as low as 60 W/cm^2^ for 561 nm (Figure 3). 

## 3. Materials and Methods 

### 3.1. Chemical Section

Commercially available reagents and solvents were used without additional purification. For column chromatography, E. Merck Kieselgel 60 was used. Thin layer chromatography (TLC) was performed on silica gel 60 F_254_ glass-backed plates (MERCK). Visualization was performed under UV light (254 or 312 nm) and staining with KMnO_4_. Synthetic routes are outlined in Scheme 2, Scheme 3 and Scheme 4.

NMR spectra were recorded on a 700 MHz Bruker Avance III NMR at 293 K. Chemical shifts are reported relative to residue peaks of CDCl_3_ (7.27 ppm for ^1^H and 77.0 ppm for ^13^C) or DMSO-d_6_ (2.51 ppm for ^1^H and 39.5 ppm for ^13^C). Melting points were measured on an SMP 30 apparatus and were uncorrected. High-resolution mass spectra (HRMS) spectra were recorded on an Agilent 6224 TOF LC/MS System (Agilent Technologies, Santa Clara, CA, USA) equipped with a dual-nebulizer ESI source and on a Bruker micrOTOF II instrument and ThermoScientific LTQ Orbitrap instrument (capillary voltage 2 kV, nanospray, needle temperature 200 °С). 

Varian Cary 100 UV/VIS Spectrophotometer and Varian Cary Eclipse Fluorescence spectrophotometer were used to measure absorption and excitation-emission spectra (5-10µM). Fluorescence quantum yields (FQY) were determined by direct comparison with Rhodamine 101 for compounds **2**, **5,** and **7**; Coumarin 153 for compound **6** and Oxazine 1 for compounds **1**, **3,** and **4** as standards [24].

### 3.2. Synthetic procedures

#### 3.2.1. (*Z*)-4-(4-(diethylamino)-2-(difluoroboryl)benzylidene)-1-methyl-2-(styryl)-1*H*-imidazol-5(4*H*)-ones (**1**) (general procedure)

(*Z*)-4-(2-(difluoroboryl)-4-(diethylamino)-benzylidene)-1,2-dimethyl-1*H*-imidazol-5(4*H*)-one [12] (319 mg, 1 mmol) and corresponding aldehyde (2 mmol) were dissolved in pyridine (3 mL) and stirred at 110 °C for 2 h. The mixture was diluted by ether (40 mL) and water (30 mL) and the crude product was separated by filtration. The precipitate was washed additionally by water (2 × 15 mL) and ether (2 × 15 mL) and dried in vacuum. Finally, the product was purified by column chromatography (CHCl_3_-EtOH 50:1).

##### (*Z*)-4-(4-(diethylamino)-2-(difluoroboryl)benzylidene)-1-methyl-2-((*E*)-2-(4-methoxyphenyl)vinyl)-1*H*-imidazol-5(4*H*)-one (**1a**)

Black solid (290 mg, 66%); mp = 255–259 °С; ^1^H NMR (DMSO-d_6_) δ 8.08 (d, *J* = 16.9 Hz, 1H), 7.75 (d, *J* = 8.8 Hz, 2H), 7.48 (s, 1H), 7.45 (d, *J* = 8.8 Hz, 1H), 7.26 (d, *J* = 16.7 Hz, 1H), 7.11 (d, *J* = 8.8 Hz, 2H), 6.90 (d, *J* = 2.0 Hz, 1H), 6.69 (dd, *J* = 8.8, 2.4 Hz, 1H), 3.86 (s, 3H), 3.52 (s, 3H), 3.49 (q, *J* = 7.2 Hz, 4H), 1.16 (t, *J* = 7.2 Hz, 6H); ^13^C NMR (DMSO-d_6_) δ 162.3, 161.9, 154.4, 150.6, 145.3, 134.6, 130.3, 129.5, 127.5, 122.2, 121.6, 114.8, 114.1, 110.8, 108.4, 55.5, 44.1, 28.3, 12.6; HRMS (ESI) *m*/*z*: 438.2166 found (calcd for C_24_H_27_BF_2_N_3_O_2_, [M + H]^+^ 438.2164).

##### (*Z*)-4-(4-(diethylamino)-2-(difluoroboryl)benzylidene)-1-methyl-2-((*E*)-2-(thiophen-2-yl)vinyl)-1*H*-imidazol-5(4*H*)-one (**1b**)

Black solid (230 mg, 56%); mp = 268–272 °С; ^1^H NMR (DMSO-d_6_) δ 8.25 (d, *J* = 16.6 Hz, 1H), 7.90 (d, *J* = 4.9 Hz, 1H), 7.66 (d, *J* = 3.2 Hz, 1H), 7.48 (s, 1H), 7.45 (d, *J* = 8.6 Hz, 1H), 7.26 (dd, *J* = 4.9, 3.6 Hz, 1H), 7.16 (d, *J* = 16.6 Hz, 1H), 6.91 (s, 1H), 6.69 (dd, *J* = 8.6, 2.2 Hz, 1H), 3.51 (s, 3H), 3.50 (q, *J* = 7.1 Hz, 4H), 1.16 (t, *J* = 7.1 Hz, 6H); ^13^C NMR (DMSO-d_6_) δ 162.2, 153.4, 150.7, 140.4, 138.1, 134.8, 133.4, 131.6, 129.8, 129.2, 122.1, 121.7, 114.1, 110.9, 109.4, 44.1, 28.4, 12.6; HRMS (ESI) *m*/*z*: 414.1624 found (calcd for C_21_H_23_BF_2_N_3_OS, [M + H]^+^ 414.1623).

##### (*Z*)-4-(4-(diethylamino)-2-(difluoroboryl)benzylidene)-2-((4-(4-methoxyphenyl)buta-1,3-dien-1-yl)-1-methyl-1*H*-imidazol-5(4*H*)-one (**1c**)

Black solid (296 mg, 64%); mp = 281–283 °С; ^1^H NMR (DMSO-d_6_) δ 8.00 (dd, *J* = 15.7, 9.5 Hz, 1H), 7.66 (d, *J* = 8.8 Hz, 2H), 7.44 (s, 1H), 7.43 (d, *J* = 8.9 Hz, 1H), 7.26–7.18 (m, 2H), 7.02 (d, *J* = 8.8 Hz, 2H), 6.90–6.87 (m, 2H), 6.69 (dd, *J* = 8.8, 2.6 Hz, 1H), 3.82 (s, 3H), 3.49 (q, *J* = 7.0 Hz, 4H), 3.45 (s, 3H), 1.16 (t, *J* = 7.0 Hz, 6H); ^13^C NMR (DMSO-d_6_) δ 162.3, 160.7, 153.9, 150.6, 147.6, 142.9, 134.5, 129.5, 129.1, 128.5, 126.4, 122.5, 121.6, 114.6, 114.1, 112.3, 110.9, 55.3, 44.1, 28.2, 12.6; HRMS (ESI) *m*/*z*: 464.2327 found (calcd for C_26_H_29_BF_2_N_3_O_2_, [M + H]^+^ 464.2321).

##### (*Z*)-4-(4-(diethylamino)-2-(difluoroboryl)benzylidene)-1-methyl-2-((*E*)-2-(pyridin-4-yl)vinyl)-1*H*-imidazol-5(4*H*)-one (**1d**)

Compound was synthesized by similar procedure. NMR spectra and other data were reported previously [17].

#### 3.2.2. (*Z*)-4-(2-(Difluoroboryl)-benzylidene)-1-methyl-2-aminoethenyl-1*H*-imidazol-5(4*H*)-ones (**2**) (general procedure)

(Z)-4-(2-(difluoroboryl)-4-(diethylamino)-benzylidene)-1,2-dimethyl-1*H*-imidazol-5(4*H*)-one [12] (319 mg, 1 mmol) and corresponding acetal [25] (3 mmol) were dissolved in DMF (10 mL) and stirred at 100 °C for 5 min. The solvent was evaporated and the product was purified by column chromatography (CHCl_3_/EtOH 50:1).

##### (*Z*)-4-(4-(diethylamino)-2-(difluoroboryl)benzylidene)-2-((*E*)-2-(dimethylamino)vinyl)-1-methyl-1*H*-imidazol-5(4*H*)-one (**2a**)

Red solid (280 mg, 75%); mp over 275 °С with decomposition; ^1^H NMR (DMSO-d_6_) δ = 8.61 (d, *J* = 12.9 Hz, 1H), 7.23 (d, *J* = 8.5 Hz, 1H), 6.91 (s, 1H), 6.83 (d, *J* = 1.5 Hz, 1H), 6.59 (dd, *J* = 8.6, 2.5 Hz, 1H), 5.16 (d, *J* = 12.5 Hz, 1H), 3.41 (q, *J* = 7.0 Hz, 4H), 3.31 (s, 3H), 3.27 (s, 3H), 3.08 (s, 3H), 1.13 (t, *J* = 7.0 Hz, 6H); ^13^C NMR (DMSO-d_6_) δ = 12.7, 27.0, 36.9, 43.8, 46.0, 77.9, 110.5, 113.6, 119.4, 121.9, 124.3, 131.1, 148.5, 156.6, 163.0; HRMS (ESI) *m*/*z*: 375.2161 found (calcd for C_19_H_25_BF_2_N_4_O, [M + H]^+^ 375.2126).

##### (*Z*)-4-(2-(difluoroboryl)-4-(diethylamino)-benzylidene)-1-methyl-2-((*E*)-2-piperidin-1-ylethenyl)-1*H*-imidazol-5(4*H*)-one (**2b**)

Red solid. Yield 364 mg, 88%; ^1^H NMR (700 MHz, DMSO-*d*_6_) δ = 8.62 (d, *J* = 12.0 Hz, 1H, CH=CH), 7.23 (d, *J* = 8.7 Hz, 1H, Ar), 6.89 (s, 1H, CH-Ar), 6.83 (d, *J* = 1.7 Hz, 1H, Ar), 6.59 (dd, *J* = 8.6, 2.5 Hz, 1H, Ar), 5.33 (d, *J* = 12.5 Hz, 1 H, CH=CH), 3.60–3.58 (m, 4H, CH_2_), 3.41 (q, *J*=7.0 Hz, 4H, CH_2_), 3.27 (s, 3H, CH_3_), 1.69–1.64 (m, 6H, CH_2_), 1.13 (t, *J* = 7.0 Hz, 6H, CH_3_); ^13^C NMR (126 MHz, DMSO-*d*_6_) δ = 12.7, 23.3, 24.9, 26.6, 27.0, 43.8, 45.5, 55.9, 77.2, 110.5, 113.6, 119.1, 121.9, 124.4, 131.0, 148.5, 155.1, 157.2, 163.0; HRMS (*m/z*) calcd. C_22_H_29_BF_2_N_4_O for [M + H]^+^: 415.2475, found: 415.2479.

##### (*Z*)-4-(2-(difluoroboryl)-4-(diethylamino)-benzylidene)-1-methyl-2-((*E*)-2-pyrrolidin-1-ylethenyl)-1*H*-imidazol-5(4*H*)-one (**2c**)

Red solid. Yield 364 mg, 91%; ^1^H NMR (700 MHz, DMSO-*d*_6_) δ = 8.78 (d, *J* = 10.0 Hz, 1H, CH=CH), 7.23 (d, *J* = 8.5 Hz, 1H, Ar), 6.90 (s, 1H, CH-Ar), 6.83 (br. s., 1H, Ar), 6.59 (dd, *J* = 8.5, 2.1 Hz, 1H, Ar), 5.09 (d, *J* = 11.6 Hz, 1H, CH=CH), 3.74 (t, *J* = 6.5 Hz, 2H, CH_2_), 3.39–3.46 (m, 6H, CH_2_), 3.27 (s, 3H, CH_3_), 2.04 (quin, *J* = 6.8 Hz, 2H, CH_2_), 1.94 (quin, *J* = 6.6 Hz, 2H, CH_2_), 1.13 (t, *J* = 6.9 Hz, 6H, CH_3_); ^13^C NMR (126 MHz, DMSO-*d*_6_) δ = 12.7, 24.3, 24.6, 26.9, 43.8, 47.1, 53.4, 78.9, 110.5, 113.6, 119.2, 122.0, 124.4, 131.1, 148.5, 152.6, 156.1, 163.0; HRMS (*m/z*) calcd. C_21_H_27_BF_2_N_4_O for [M + H]^+^: 401.2319, found: 401.2310.

##### (*Z*)-4-(4-(diethylamino)-2-(difluoroboryl)benzylidene)-1-methyl-2-((*E*)-2-morpholinovinyl)-1*H*-imidazol-5(4*H*)-one (**2d**)

Red solid (354 mg, 85%); mp over 275 °С with decomposition; ^1^H NMR (DMSO-d_6_) δ = 8.59 (d, *J* = 12.9 Hz, 1H), 7.25 (d, *J* = 8.5 Hz, 1H), 6.94 (s, 1H), 6.83 (d, *J* = 2.1 Hz, 1H), 6.59 (dd, *J* = 8.5, 2.6 Hz, 1H), 5.39 (d, *J* = 12.9 Hz, 1H), 3.74–3.72 (m, 4H), 3.64–3.62 (m, 4H), 3.41 (q, *J* = 7.0 Hz, 4H), 3.27 (s, 3H), 1.13 (t, *J* = 7.0 Hz, 6H); ^13^C NMR (DMSO-d_6_) δ = 12.6, 27.1, 43.8, 45.2, 54.1, 65.2, 66.5, 77.9, 110.5, 113.6, 120.1, 121.9, 124.2, 131.3, 148.7, 155.1, 157.2, 162.9; HRMS (ESI) *m*/*z*: 417.2271 found (calcd for C_21_H_27_BF_2_N_4_O_2_, [M + H]^+^ 417.2268).

#### 3.2.3. (*Z*)-4-(4-(Diethylamino)-2-(difluoroboryl)-benzylidene)-1*H*-imidazo [1,2-a]pyridin-5(4*H*)-one (**3**)

Compound was synthesized as reported previously [17].

#### 3.2.4. 7-(Diethylamino)-3*H*-phenoxazin-3-one (**4**)

3-(diethylamino)phenol (1.65 g, 10 mmol) and 4-nitrosobenzene-1,3-diol (1,39 g, 10 mmol) were dissolved in acetonitrile (90 mL). The sulfuric acid (10 mL) was added upon stirring and the mixture was heated to 60 °C for 5 hours. The mixture was diluted by water (100 mL) and neutralized by addition of the K_2_CO_3_ (pH = 7–9). The product was extracted by EtOAc (3 × 150 mL) and the solution was washed with water (2 × 150 mL) and brine (2 × 150 mL) and dried over Na_2_SO_4_. The solvent was evaporated and the product was purified by column chromatography (EtOAc-hexane 3:2).

Red solid (590 mg, 22%); mp = 187–189 °С; ^1^H NMR (DMSO-d_6_) δ 7.58 (d, *J* = 9.3 Hz, 1H), 7.45 (d, *J* = 9.5 Hz, 1H), 6.91 (dd, *J* = 9.3, 2.5 Hz, 1H), 6.68 (d, *J* = 2.5 Hz, 1H), 6.64 (dd, *J* = 9.5, 2.0 Hz, 1H), 6.16 (d, *J* = 2.0 Hz, 1H), 3.54 (q, *J* = 7.3 Hz, 4H), 1.17 (t, *J* = 7.1 Hz, 6H); ^13^C NMR (DMSO-d_6_) δ 184.1, 152.0, 150.0, 146.6, 139.4, 134.2, 131.7, 130.9, 126.1, 111.4, 104.7, 95.8, 44.7, 12.4; HRMS (ESI) *m*/*z*: 269.1283 found (calcd for C_16_H_17_N_2_O_2_, [M + H]^+^ 269.1290).

#### 3.2.5. (*Z*)-2-acetyl-4-(4-(diethylamino)benzylidene)-1-methyl-1*H*-imidazol-5(4*H*)-one (**5**)

(*Z*)-4-(4-(diethylamino)benzylidene)-2-ethyl-1-methyl-1*H*-imidazol-5(4*H*)-one [4] (2.85 g, 10 mmol) was dissolved in dioxane (100 mL), selenium dioxide (5.55 g, 50 mmol) was added, and the mixture was refluxed for 5 min. The solvent was removed on rotary evaporator. The mixture was dissolved by EtOAc (400 mL), washed with water (2 × 150 mL) and brine (2 × 150 mL), and dried over Na_2_SO_4_. The solvent was evaporated and the product was purified by column chromatography (CHCl_3_-EtOH 50:1).

Red solid (1.20 g, 40%); mp = 144–147 °С; ^1^H NMR (DMSO-d_6_) δ 8.20–8.10 (bs, 2H), 7.30 (s, 1H), 6.80 (d, *J* = 9.1 Hz, 2H), 3.47 (q, *J* = 7.1 Hz, 4H), 3.29 (s, 3H), 2.59 (s, 3H), 1.14 (t, *J* = 7.1 Hz, 6H); ^13^C NMR (DMSO-d_6_) δ 192.3, 169.1, 150.9, 150.5, 136.1, 135.3, 132.7, 120.7, 111.6, 44.1, 28.2, 26.0, 12.5; HRMS (ESI) *m*/*z*: 300.1712 found (calcd for C_17_H_22_N_3_O_2_, [M + H]^+^ 300.1712).

#### 3.2.6. (*Z*)-4-(4-(diethylamino)-2-hydroxybenzylidene)-1,2-dimethyl-1*H*-imidazol-5(4*H*)-one (**6**)

The 4-(diethylamino)-2-hydroxybenzaldehyde (2 mmol) was dissolved in CHCl_3_ (20 mL) and mixed with methylamine solution (40% aqueous, 1.0 mL) and anhydrous Na_2_SO_4_ (5 g). The mixture was stirred for 48 h at room temperature, filtered and dried over the additional Na_2_SO_4_. The solvent was evaporated, the residue was added to ethyl((1-methoxy)amino)acetate (450 mg g, 2.8 mmol). The mixture was stirred for 24 h at room temperature, and solvents were evaporated and the product was purified by column chromatography (CHCl_3_/EtOH 100:1).

Orange solid (330 mg, 58%); mp = 212–217 °С; ^1^H NMR (DMSO-d_6_) δ 7.62–7.55 (bs, 1H), 7.04 (s, 1H), 6.28 (dd, *J* = 8.8, 2.4 Hz, 1H), 6.05 (d, *J* = 2.4 Hz, 1H), 3.37 (q, *J* = 7.0 Hz, 4H), 3.11 (s, 3H), 2.33 (s, 3H), 1.11 (t, *J* = 7.0 Hz, 6H); ^13^C NMR (DMSO-d_6_) δ 167.4, 160.0, 155.9, 151.9, 137.2, 128.8, 126.9, 108.8, 104.4, 98.1, 43.8, 26.2, 14.8, 12.6; HRMS (ESI) *m*/*z*: 288.1709 found (calcd for C16H22N3O2, [M + H]^+^ 288.1712).

#### 3.2.7. (*Z*)-2-(4-(diethylamino)benzylidene)-6,7-dihydroimidazo[1,2-a] pyrazine-3,8(2*H*,5*H*)-dione (**7**)

The compound **7** was synthesized as reported previously [20].

### 3.3. Plasmids

We used previously published [4] mammalian expression vectors encoding H2B-TagBFP-lipocalin mutants DiB1 (A27C, L132N), DiB2 (A27C), DiB3 (V65F, L132Q), Blc-L141Q, Blc-V74F, cytokeratin-18-DiB1, and alpha-actinin-TagBFP-DiB1. 

### 3.4. Chromophore Titration

To investigate the binding of compound **3** to Blc-mutant proteins, fluorescence titration was performed. The data were obtained using newly purified and dialyzed protein, and newly diluted fluorophore solutions. 

Samples of dry compound **3** were diluted in DMSO. This solution was used for the subsequent preparation of the 1000× stock solution in 96% EtOH. The concentration of the compound **3** in the stock solution was measured using Cary 100 UV/VIS spectrophotometer, and the 100× working solution in PBS buffer was made before each titration. 

The concentration of the proteins was evaluated using sample absorption at 280 nm and extinction coefficients computed by the ProtParam tool (http://web.expasy.org/protparam/). To reduce dilution-induced artifacts during titration, series of diluted protein solutions were also prepared. 

The compound **3** solution (10 μL) and required an amount of protein solution and PBS up to 1 mL were added into a cuvette, gently stirred, and immediately measured using Varian Cary Eclipse fluorescence spectrophotometer. For each compound **3**-protein pair points with at least two different fluorophore and fifteen protein concentrations were measured. 

### 3.5. Analysis of Fluorescence Titration Data

The fluorescence titration data were fitted to the 1:1 binding model. Supposing Blc-mutant proteins as monomers with a single binding site for fluorophore, the binding of the fluorophore to a Blc-mutant protein can be described as P + F ⇌ PF, where P is free protein, F is a free fluorophore, and PF is protein-fluorophore complex. Then the dissociation constant (K_d_) equation is:

$$K_{d}=\frac{{\left[P\right]}_{\mathrm{eq}}{\left[F\right]}_{\mathrm{eq}}}{{\left[\mathrm{PF}\right]}_{\mathrm{eq}}},$$

where [P]_eq_ is free protein, [F]_eq_ is free fluorophore, and [PF]_eq_ is protein-fluorophore concentrations at equilibrium.

As we knew only the initial concentration of the protein and the fluorophore, and had not any tool for the free protein and the free fluorophore concentration at equilibrium measurement, the K_d_ equation was rewritten using mass balance equations:

[P]_init_ = [P]_eq_ + [PF]_eq_

[F]_init_ = [F]_eq_ + [PF]_eq_

where [P]_init_ and [F]_init_ are initial concentration of the protein and the fluorophore, respectively:

[P]_eq_ = [P]_init_ − [PF]_eq_

[F]_eq_ = [F]_init_ − [PF]_eq_

$$K_{d}=\frac{\left({\left[P\right]}_{\mathrm{init}}-{\left[\mathrm{PF}\right]}_{\mathrm{eq}}\right)\left({\left[F\right]}_{\mathrm{init}}-{\left[\mathrm{PF}\right]}_{\mathrm{eq}}\right)}{{\left[\mathrm{PF}\right]}_{\mathrm{eq}}}$$

$$\left({\left[P\right]}_{\mathrm{init}}-{\left[\mathrm{PF}\right]}_{\mathrm{eq}}\right)\left({\left[F\right]}_{\mathrm{init}}-{\left[\mathrm{PF}\right]}_{\mathrm{eq}}\right)=K_{d}{\left[\mathrm{PF}\right]}_{\mathrm{eq}}$$

$${\left[P\right]}_{\mathrm{init}}{\left[F\right]}_{\mathrm{init}}-{\left[\mathrm{PF}\right]}_{\mathrm{eq}}{\left[F\right]}_{\mathrm{init}}-{\left[P\right]}_{\mathrm{init}}{\left[\mathrm{PF}\right]}_{\mathrm{eq}}+{\left[\mathrm{PF}\right]}_{\mathrm{eq}}^{2}-K_{d}{\left[\mathrm{PF}\right]}_{\mathrm{eq}}=0$$

$${\left[\mathrm{PF}\right]}_{\mathrm{eq}}^{2}-{\left[\mathrm{PF}\right]}_{\mathrm{eq}}\left({\left[F\right]}_{\mathrm{init}}+{\left[P\right]}_{\mathrm{init}}+K_{d}\right)+{\left[P\right]}_{\mathrm{init}}{\left[F\right]}_{\mathrm{init}}=0$$

The protein-fluorophore complex ([PF]_eq_) concentration can be expressed as:

(1) $${\left[\mathrm{PF}\right]}_{\mathrm{eq}}=\frac{\left({\left[F\right]}_{\mathrm{init}}+{\left[P\right]}_{\mathrm{init}}+K_{d}\right)-\sqrt{{\left({\left[F\right]}_{\mathrm{init}}+{\left[P\right]}_{\mathrm{init}}+K_{d}\right)}^{2}-4{\left[P\right]}_{\mathrm{init}}{\left[F\right]}_{\mathrm{init}}}}{2}$$

Thus, the fluorescence emission intensity (F) of the protein-fluorophore solution can be expressed as:

(2) $$F={\left[\mathrm{PF}\right]}_{\mathrm{eq}}\times \Phi_{\left[\mathrm{PF}\right]}+\left({\left[F\right]}_{\mathrm{init}}-{\left[\mathrm{PF}\right]}_{\mathrm{eq}}\right)\times \Phi_{\left[F\right]}$$

where Φ_[PF]_ and Φ_[F]_ are the relative fluorescence quantum yields of the protein-fluorophore complex and the pure fluorophore, respectively.

Therefore, to determine apparent K_d_, the experimentally obtained data were fitted to equation 2 by *leastsq* method from Scipy *optimize* package.

### 3.6. Cell Culture 

HEK293T or HeLa cells were grown in Dulbecco’s modification of Eagle’s medium (DMEM) (PanEco, Moscow, Russia) containing 50 U/mL penicillin and 50 µg/mL streptomycin (PanEco), 2 mM l-glutamine (PanEco), and 10% fetal bovine serum (HyClone, Thermo Scientific, Waltham, MA, USA) at 37 °C and 5% CO_2_. For background and membrane fluorescence screening of chromophores, the day before experiment cells were straight plated in 8-Well µ-Slide (IBIDI, Martinsried, Germany). For protein–chromophore complex fluorescence screening the day before, transfection cells were plated in 35 mm cell culture dishes. For transient transfections, the FuGENE 6 transfection reagent (Roche, Basel, Switzerland) was used according to protocol. The day before experiment transfected cells were plated in µ-Slide 8 Well. 

### 3.7. Fluorescence Microscopy

Before imaging, cells were washed with PBS and placed in minimum essential medium Eagle (MEM) (Sigma-Aldrich, St. Louis, MO, USA) supplemented with 20 mM HEPES (PanEco).

Widefield fluorescence microscopy for measurement of background fluorescence and membrane fluorescence of fluorogenic dye was performed with the BZ-9000 fluorescence microscope (Keyence) equipped with 40× dry and 63× oil-immersion objectives (filter sets OP-79304 DAPI-BP, OP-79301 SB GFP-BP, OP-79302 SB TexasRed, and OP-79305 CY5).

Single-molecule localization super-resolution imaging of living cells was carried out on a Nikon Eclipse Ti N-STORM microscope (Nikon, Japan) controlled by NIS-Elements Software. The excitation light from 561 nm (60 W/cm^2^) laser lines was focused on the sample with a 100× oil-immersion objective (Apo TIRF/1.49, Nikon) and PFS (perfect focus system). Super-resolution images were rendered with ThunderSTORM software [26].

## 4. Conclusions

To conclude, we have developed novel fluorogenic compounds for live-cell protein labeling with lipocalin-based protein hosts. In comparison with previously published fluorogen **M739**, the emission of the compound **3** in complex with the lipocalin DiB1 is considerably shifted to the red region of the spectrum (625 nm for **3** vs. 542 nm for **M739**). The brightness of the proposed tag is close to that of the fluorescent protein mCherry. Unlike the fluorescent proteins, the fluorogen–protein pair does not need oxygen and time for maturation and can be switched on and off at will by adding or removing of the fluorogen. Expansion of the color range of suitable fluorogens for the same protein host has been shown to be useful for color multiplexing in live-cell imaging [27]. On-demand labeling with red-shifted fluorogens holds the promise for lowering the phototoxicity in light-intense applications, such as nanoscopy and light sheet microscopy. 

## Figures and Tables

**Table 1:** Photophysical properties of the protein–fluorogen bipartite tags.

Fluorescent Tag	λ_abs_, nm	λ_em_, nm	*EC*	*K*_d_, µM	FQY
DiB1:**3**	605	625	55	0.09	0.29
DiB2:**3**	600	625	55	0.54	-
DiB3:**3**	622	637	55	2.39	-
Blc-L141Q:**3**	607	630	55	0.12	-
Blc-V74F:**3**	605	634	55	0.48	-
DiB1:**M739** [4]	513	542	45.8	0.1	0.32
DiB2:**M739** [4]	510	539	51	4	0.32
DiB3:**M739** [4]	546	565	43	9	0.15
mCherry, data from Ref. [23]	586	610	88	N/A	0.23

λ_abs_—the wavelength of maximal absorption; λ_em_—the wavelength of maximal emission; *EC*—the molar absorption coefficient at λ_abs_ (mM·cm^−1^); *K*_d_—the dissociation constant; FQY—fluorescence quantum yield.

## Supplementary Materials

Supplementary materials can be found at http://www.mdpi.com/1422-0067/19/12/3778/s1.

## References

[1] Paige J.S., Wu K.Y., Jaffrey S.R. (2011). RNA mimics of green fluorescent protein. Science.

[2] Szent-Gyorgyi C., Schmidt B.F., Schmidt B.A., Creeger Y., Fisher G.W., Zakel K.L., Adler S., Fitzpatrick J.A.J., Woolford C.A., Yan Q. (2008). Fluorogen-activating single-chain antibodies for imaging cell surface proteins. Nat. Biotechnol..

[3] Plamont M.-A., Billon-Denis E., Maurin S., Gauron C., Pimenta F.M., Specht C.G., Shi J., Quérard J., Pan B., Rossignol J. (2016). Small fluorescence-activating and absorption-shifting tag for tunable protein imaging in vivo. Proc. Natl. Acad. Sci. USA.

[4] Bozhanova N.G., Baranov M.S., Klementieva N.V., Sarkisyan K.S., Gavrikov A.S., Yampolsky I.V., Zagaynova E.V., Lukyanov S.A., Lukyanov K.A., Mishin A.S. (2017). Protein labeling for live cell fluorescence microscopy with a highly photostable renewable signal. Chem. Sci..

[5] Dou J., Vorobieva A.A., Sheffler W., Doyle L.A., Park H., Bick M.J., Mao B., Foight G.W., Lee M.Y., Gagnon L.A. (2018). De novo design of a fluorescence-activating β-barrel. Nature.

[6] Raue R. (2000). Methine Dyes and Pigments. Ullmann’s Encyclopedia of Industrial Chemistry.

[7] Paul W. (2000). Xanthene Dyes. Kirk-Othmer Encyclopedia of Chemical Technology.

[8] Loudet A., Burgess K. (2007). BODIPY dyes and their derivatives: Syntheses and spectroscopic properties. Chem. Rev..

[9] Christie R.M. (2008). Fluorescent dyes. Rev. Prog. Color. Relat. Top..

[10] Walker C.L., Lukyanov K.A., Yampolsky I.V., Mishin A.S., Bommarius A.S., Duraj-Thatte A.M., Azizi B., Tolbert L.M., Solntsev K.M. (2015). Fluorescence imaging using synthetic GFP chromophores. Curr. Opin. Chem. Biol..

[11] Feng G., Luo C., Yi H., Yuan L., Lin B., Luo X., Hu X., Wang H., Lei C., Nie Z. (2017). DNA mimics of red fluorescent proteins (RFP) based on G-quadruplex-confined synthetic RFP chromophores. Nucleic Acids Res..

[12] Baranov M.S., Solntsev K.M., Baleeva N.S., Mishin A.S., Lukyanov S.A., Lukyanov K.A., Yampolsky I.V. (2014). Red-Shifted Fluorescent Aminated Derivatives of a Conformationally Locked GFP Chromophore. Chem.-A Eur. J..

[13] Baleeva N.S., Zaitseva S.O., Gorbachev D.A., Smirnov A.Y., Zagudaylova M.B., Baranov M.S. (2017). The Role of N -Substituents in Radiationless Deactivation of Aminated Derivatives of a Locked GFP Chromophore. Eur. J. Org. Chem..

[14] Grabowski Z.R., Rotkiewicz K., Rettig W. (2003). Structural changes accompanying intramolecular electron transfer: Focus on twisted intramolecular charge-transfer states and structures. Chem. Rev..

[15] Subach F.V., Piatkevich K.D., Verkhusha V.V. (2011). Directed molecular evolution to design advanced red fluorescent proteins. Nat. Methods.

[16] Song W., Filonov G.S., Kim H., Hirsch M., Li X., Moon J.D., Jaffrey S.R. (2017). Imaging RNA polymerase III transcription using a photostable RNA-fluorophore complex. Nat. Chem. Biol..

[17] Baleeva N.S., Myannik K.A., Yampolsky I.V., Baranov M.S. (2015). Bioinspired Fluorescent Dyes Based on a Conformationally Locked Chromophore of the Fluorescent Protein Kaede. Eur. J. Org. Chem..

[18] Otsuki S., Taguchi T. (1997). Solvent-dependent photophysical properties of aminophenoxazone dyes as optical probes. J. Photochem. Photobiol. A Chem..

[19] Greenspan P., Mayer E.P., Fowler S.D. (1985). Nile red: A selective fluorescent stain for intracellular lipid droplets. J. Cell Biol..

[20] Baleeva N.S., Levina E.A., Baranov M.S. (2017). Synthesis of 2-arylidene-6,7-dihydroimidazo[1,2-a]pyrazine-3,8(2H,5H)-diones by oxidation of 4-arylidene-2-methyl-1H-imidazol-5(4H)-ones with selenium dioxide. Chem. Heterocycl. Compd..

[21] Yampolsky I.V., Remington S.J., Martynov V.I., Potapov V.K., Lukyanov S., Lukyanov K.A. (2005). Synthesis and properties of the chromophore of the asFP595 chromoprotein from Anemonia sulcata. Biochemistry.

[22] Baldridge A., Feng S., Chang Y.-T., Tolbert L.M. (2011). Recapture of GFP chromophore fluorescence in a protein host. ACS Comb. Sci..

[23] Bindels D.S., Haarbosch L., van Weeren L., Postma M., Wiese K.E., Mastop M., Aumonier S., Gotthard G., Royant A., Hink M.A. (2017). mScarlet: A bright monomeric red fluorescent protein for cellular imaging. Nat. Methods.

[24] Würth C., Grabolle M., Pauli J., Spieles M., Resch-Genger U. (2013). Relative and absolute determination of fluorescence quantum yields of transparent samples. Nat. Protoc..

[25] Yao B., Zhang Y., Li Y. (2010). Copper-catalyzed coupling reaction of C-OMe bonds adjacent to a nitrogen atom with terminal alkynes. J. Org. Chem..

[26] Ovesný M., Křížek P., Borkovec J., Svindrych Z., Hagen G.M. (2014). ThunderSTORM: A comprehensive ImageJ plug-in for PALM and STORM data analysis and super-resolution imaging. Bioinformatics.

[27] Li C., Plamont M.-A., Sladitschek H.L., Rodrigues V., Aujard I., Neveu P., Le Saux T., Jullien L., Gautier A. (2017). Dynamic multicolor protein labeling in living cells. Chem. Sci..

