# Supplementary material for: Red-Shifted Aminated Derivatives of GFP Chromophore for Live-Cell Protein Labeling with Lipocalins
Source: Int J Mol Sci. 2018 Nov 28;19(12):3778. doi: 10.3390/ijms19123778 (PMC6320917; doi:10.3390/ijms19123778)
Supplement: Supplementary file 1 [file ijms-19-03778-s001.pdf]

Supplementary information

Red-shifted aminated derivatives of GFP chromophore for live-cell protein labelling with lipocalins

Nina G. Bozhanova, Mikhail S. Baranov, Nadezhda S. Baleeva, Alexey S. Gavrikov and Alexander S. Mishin

Supplementary figures and tables

Table S1	2
Figure S1	3
NMR spectra	4-23

Table S1. Optical properties of synthesized compounds.

		**1a**	**1b**	**1c**	**1d**	**2a**	**2c**	**2b**	**2d**	**3**	**4**	**5**	**6**	**7**	**M739**
water	Abs[a]	602 (-)[c]	600 (-)[c]	609 (-)[c]	597 (31500)	532 (32000)	534 (31000)	529 (30500)	539 (33500)	603 (55000)	608 (58500)	529 (31000)	465 (39000)[a]	532 (56500)	520 (53500)
	Em[b]	690 (1.2)	695 (0.6)	710 (0.5)	705 (0.3)	597 (60)	599 (50)	600 (58)	607 (13)	626 (12)	632 (5.1)	615 (0.2)	516 (0.1)[b]	581 (0.4)	563 (3.1)
MeOH	Abs	578 (37000)	587 (35500)	592 (44500)	597 (33500)	525 (41000)	528 (40500)	526 (42000)	529 (45000)	596 (58000)	580 (53000)	516 (33500)	455 (48000)	503 (52500)	505 (48000)
	Em	657(8.6)	678 (3.9)	670 (1.7)	697 (1.5)	591(67)	594 (61)	591 (89)	597 (56)	635 (37)	626 (15)	608 (0.2)	501 (0.3)	576 (0.3)	557 (22)
CH_3CN	Abs	567 (33000)	580 (33000)	587 (38000)	582 (30000)	512 (40500)	514 (40500)	512 (41500)	517 (43500)	580 (52000)	545 (45000)	503 (36000)	443 (43000)	488 (49500)	494 (45500)
	Em	677(6.4)	708 (2.1)	715 (1.1)	716 (1.6)	582 (60)	581 (58)	582 (60)	591 (51)	639 (40)	610 (87)	601 (0.2)	492 (0.3)	562 (0.2)	554 (31)
EtOAc	Abs	565 (34000)	579 (32000)	584 (38000)	582 (30500)	514 (42500)	517 (38500)	514 (39000)	518 (44000)	583 (52000)	532 (44000)	496 (37000)	440 (45000)	473 (48500)	492 (47000)
	Em	660 (12)	690 (3.6)	703 (1.8)	702 (2.7)	570 (63)	567 (64)	572 (82)	580 (53)	631 (53)	592 (55)	578 (0.1)	483 (0.4)	543 (0.2)	538 (63)
dioxane	Abs	571 (33500)	583 (31000)	589 (35500)	591 (31000)	519 (40500)	523 (39500)	521 (39500)	524 (43500)	589 (55000)	534 (40000)	495 (33000)	446 (42000)	478 (50500)	497 (51000)
	Em	655 (16)	685 (4.9)	701 (2.6)	696 (4.8)	569 (70)	568 (70)	570 (90)	576 (53)	624 (66)	587 (30)	572 (0.1)	480 (0.5)	541 (0.2)	538 (72)

a – peak maximum in nm (extinction coefficient in $(M\ cm)^{-1}$), b – peak maximum in nm (fluorescence quantum yield in %), c - non soluble enough to measure EC correctly

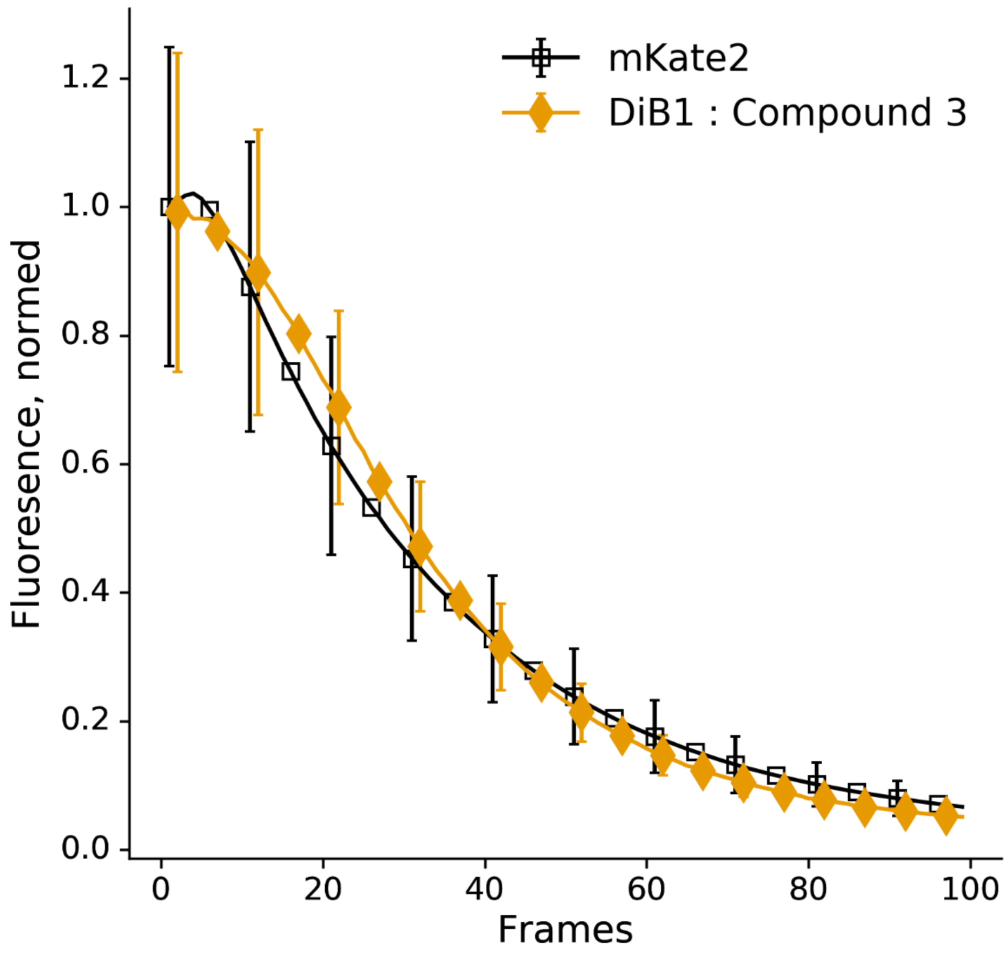

Figure S1. Photostability of DiB1:3 complex in living cells. (Orange line) - photobleaching curves of HEK293T expressing H2B fused with DiB1 and stained with compound **3** in a widefield setup. (black line) - curve for H2B-mKate2 under the same imaging conditions. HEK293T cells expressing H2B fused with the corresponding reporter were imaged 24h after transient transfection in MEM media supplemented with 20mM HEPES. Data points acquired with 40x 0.85NA objective lens at 10s intervals, with 8s bursts of 40 W/cm^2 green light (pE300 light source, CoolLED) in between the imaging frames. Mean values and standard deviation (n=21 cells) are depicted. Markers are shown for every 5 data points for clarity.

Copies of ^{1}H and ^{13}C NMR spectra

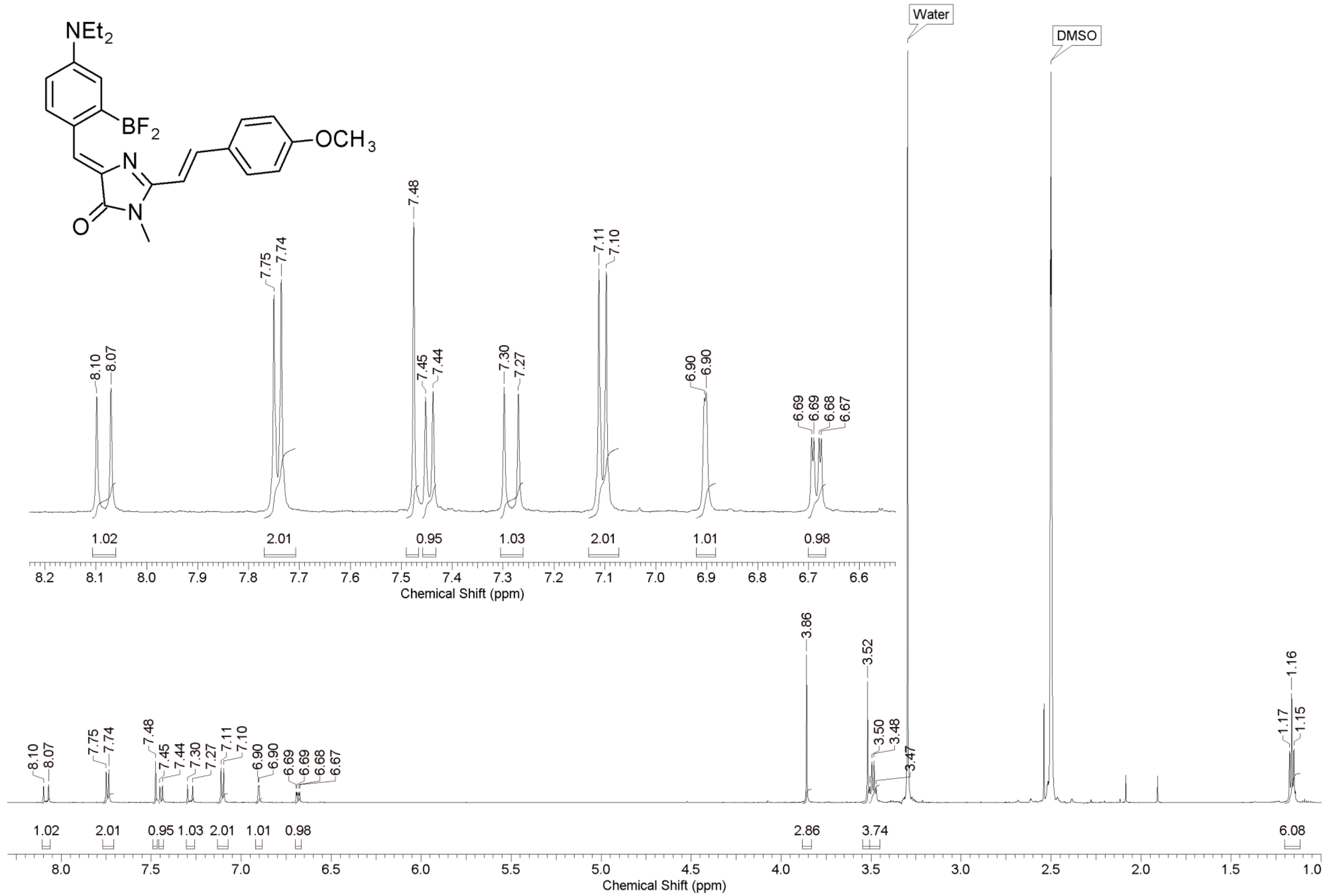

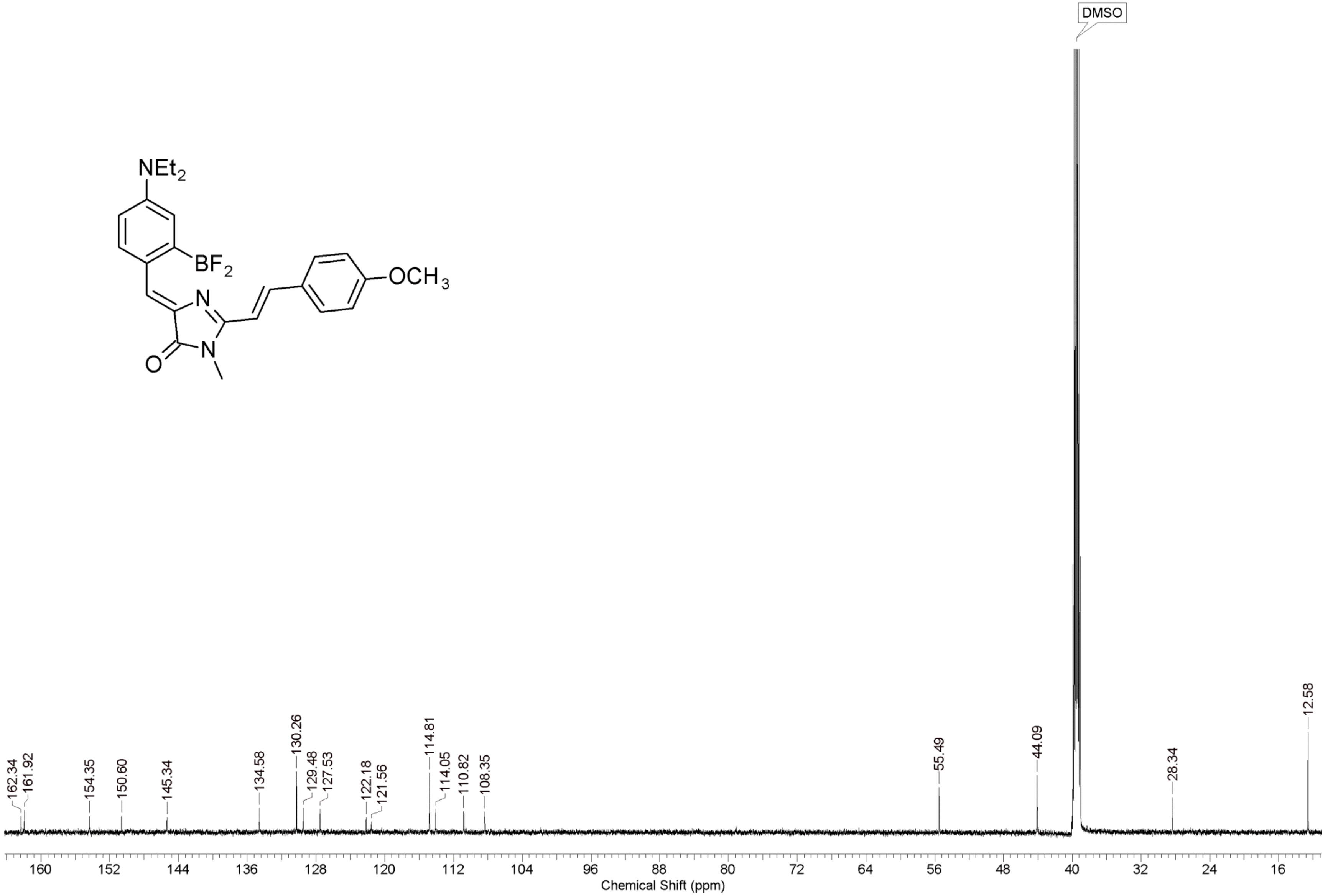
NEt2
BF2
OCH3
N
N
O
DMSO
162.34
161.92
154.35
150.60
145.34
134.58
130.26
129.48
127.53
122.18
121.56
114.81
114.05
110.82
108.35
55.49
44.09
28.34
12.58
160
152
144
136
128
120
112
104
96
88
80
72
64
56
48
40
32
24
16
Chemical Shift (ppm)

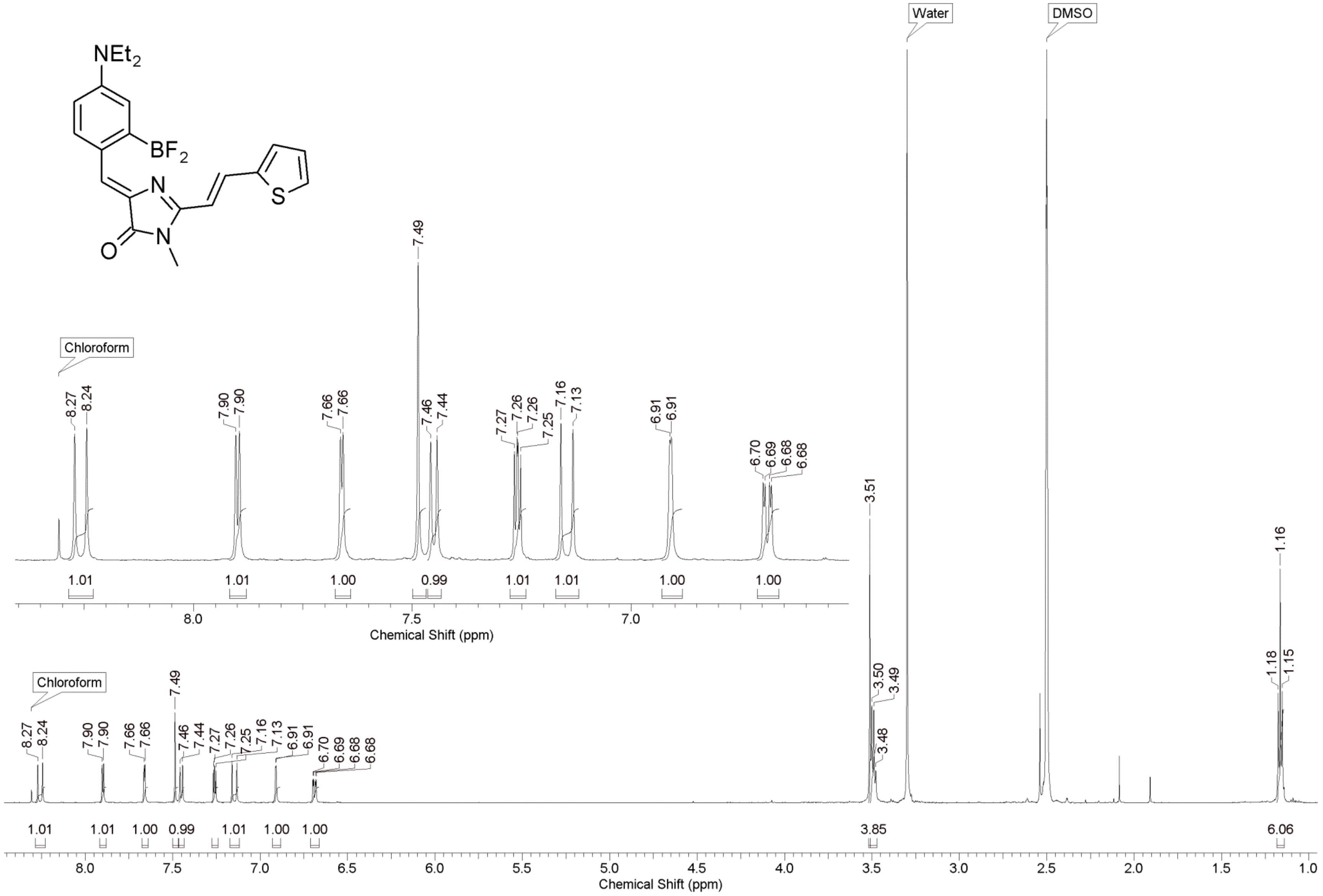

NEt2
BF2
N
N
O
S
Water
DMSO
Chloroform
8.27
8.24
7.90
7.90
7.66
7.66
7.49
7.46
7.44
7.27
7.26
7.26
7.25
7.16
7.13
6.91
6.91
6.70
6.69
6.68
6.68
1.01
1.01
1.00
0.99
1.01
1.01
1.00
1.00
Chemical Shift (ppm)
3.51
3.50
3.49
3.48
1.18
1.16
1.15
3.85
6.06

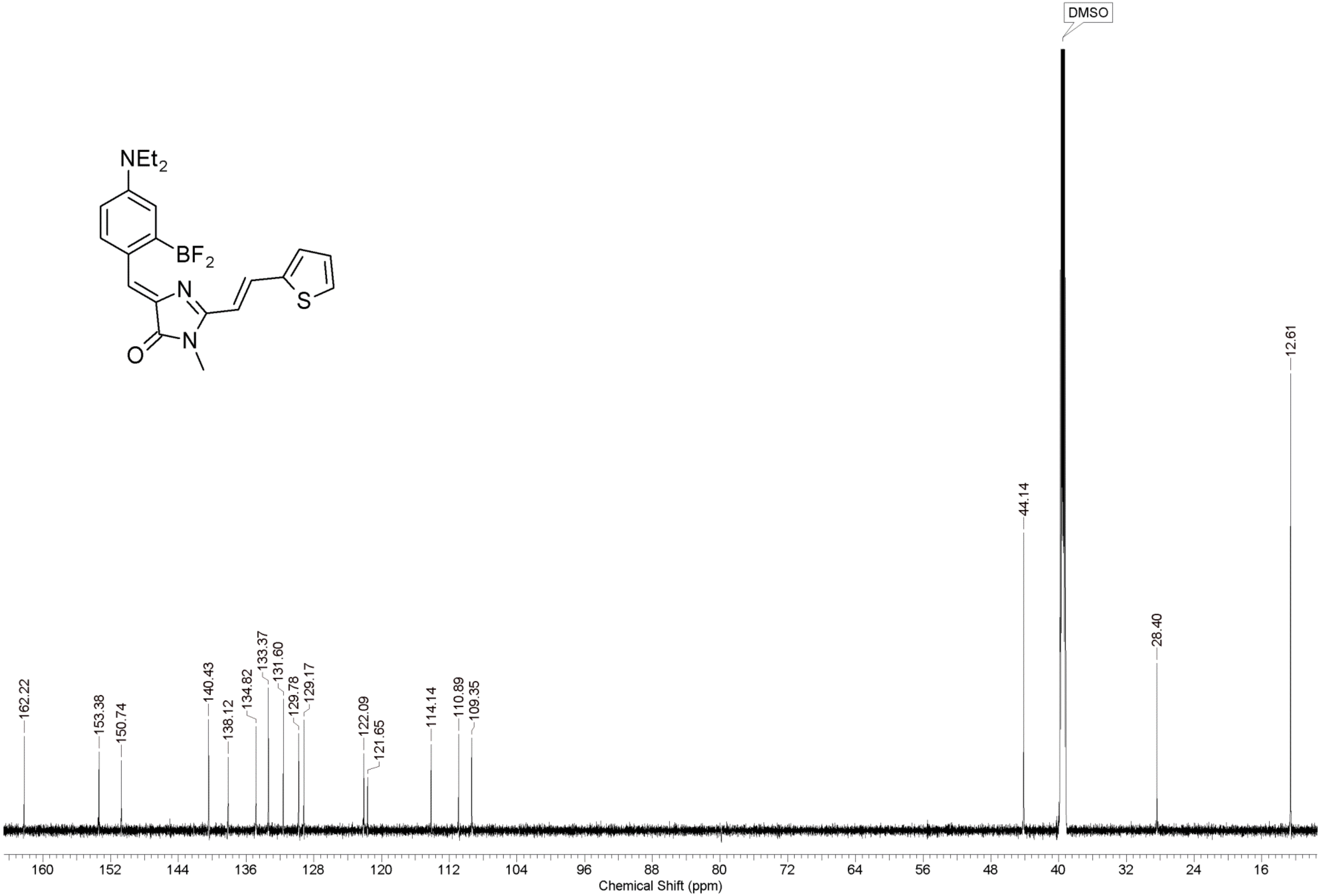
NEt2
BF2
N
S
N
O
DMSO
162.22
153.38
150.74
140.43
138.12
134.82
133.37
131.60
129.78
129.17
122.09
121.65
114.14
110.89
109.35
44.14
28.40
12.61
160
152
144
136
128
120
112
104
96
88
80
72
64
56
48
40
32
24
16
Chemical Shift (ppm)

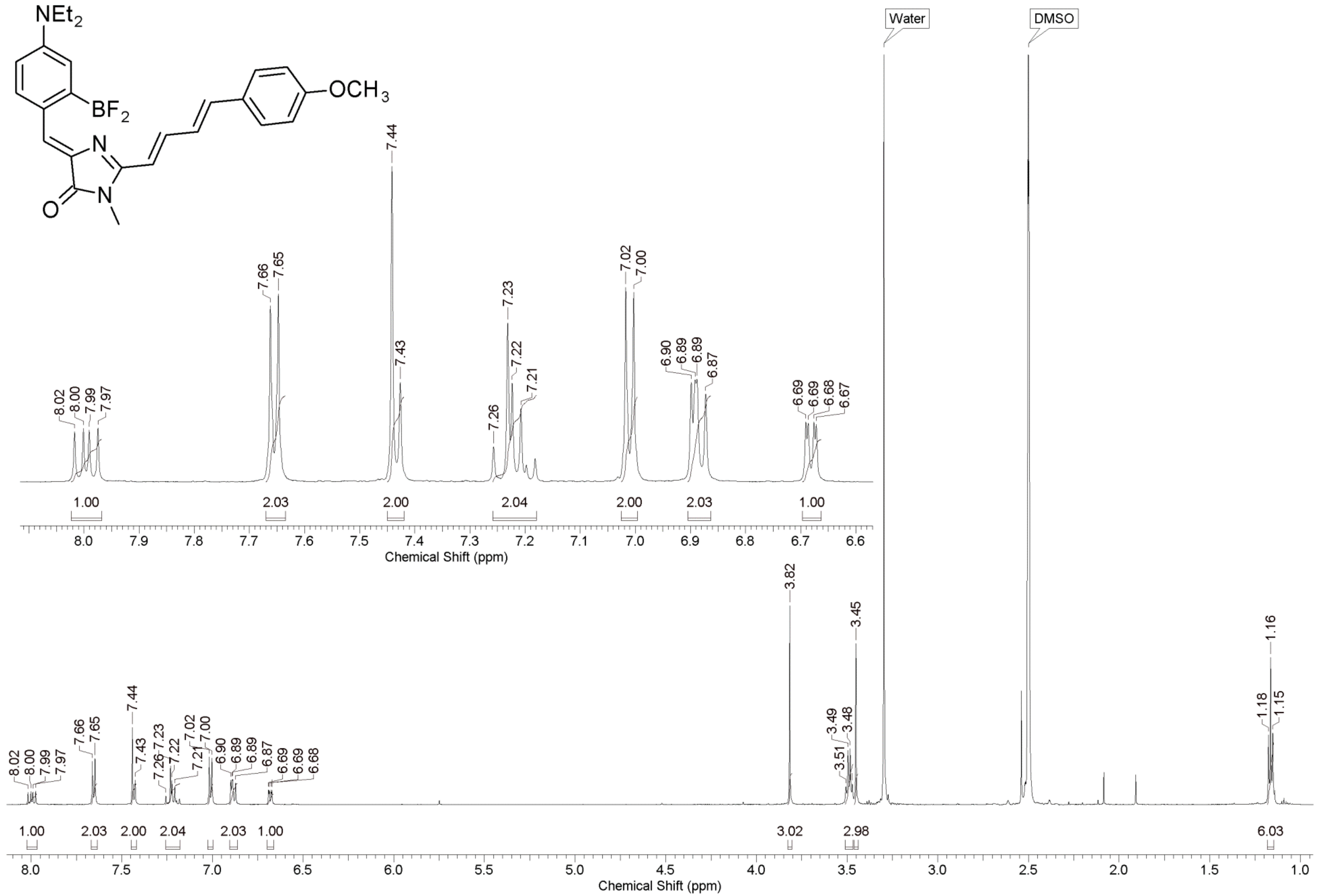
NEt2
BF2
OCH3
N
N
O
Water
DMSO
8.02 8.00 7.99 7.97
7.66 7.65
7.44 7.43
7.26 7.23 7.22 7.21
7.02 7.00
6.90 6.89 6.89 6.87
6.69 6.69 6.68 6.67
1.00 2.03 2.00 2.04 2.00 2.03 1.00
Chemical Shift (ppm)
3.82
3.51 3.49 3.48 3.45
1.18 1.16 1.15
3.02 2.98 6.03
Chemical Shift (ppm)

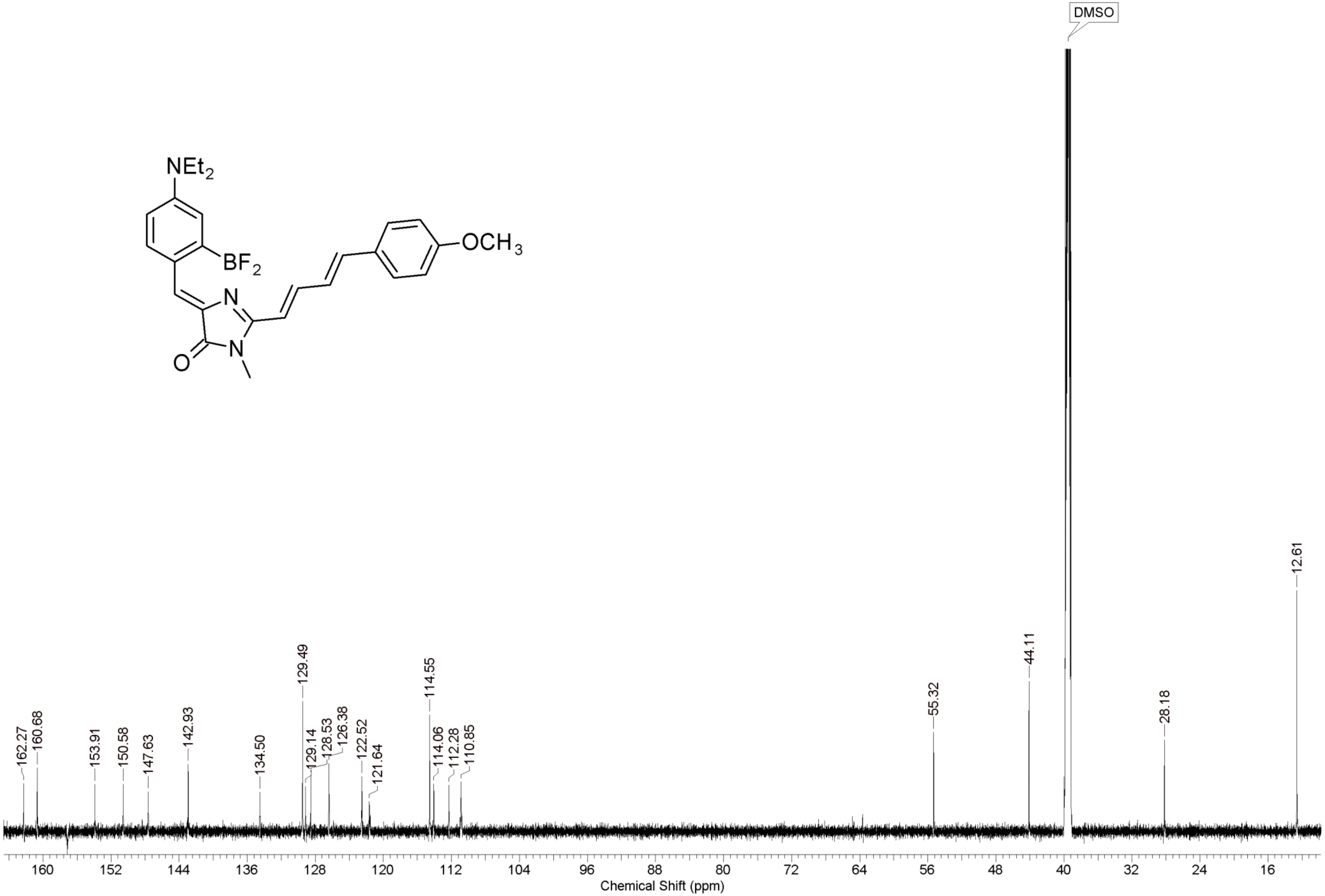
NEt2
BF2
OCH3
N
N
O
DMSO
162.27
160.68
153.91
150.58
147.63
142.93
134.50
129.49
129.14
128.53
126.38
122.52
121.64
114.55
114.06
112.28
110.85
55.32
44.11
28.18
12.61
160
152
144
136
128
120
112
104
96
88
80
72
64
56
48
40
32
24
16
Chemical Shift (ppm)

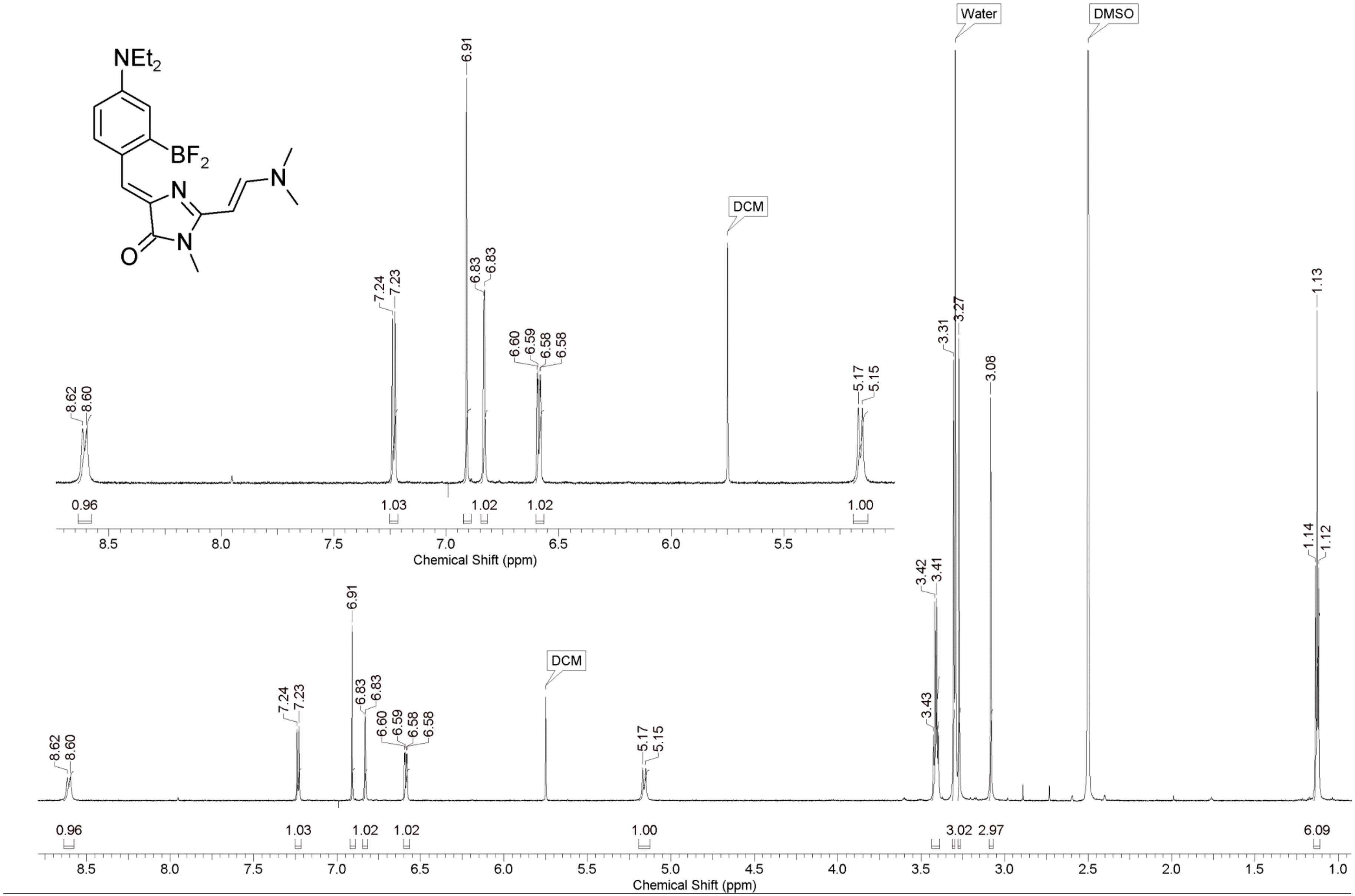
NEt2
BF2
N
N
N
O
Water
DMSO
DCM
DCM
Chemical Shift (ppm)
Chemical Shift (ppm)

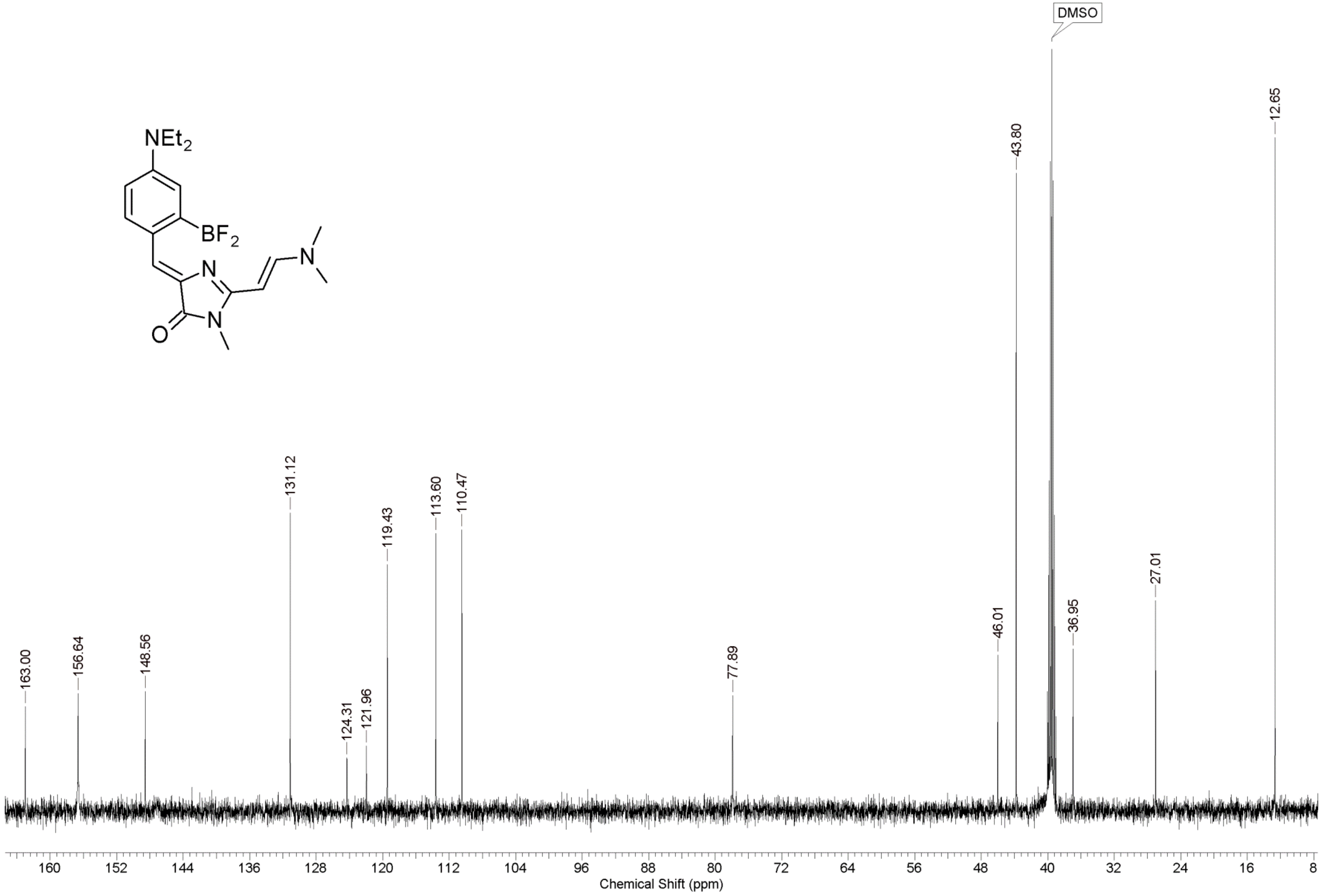

NEt2
BF2
N
N
O
DMSO
163.00
156.64
148.56
131.12
124.31
121.96
119.43
113.60
110.47
77.89
46.01
43.80
36.95
27.01
12.65
160
152
144
136
128
120
112
104
96
88
80
72
64
56
48
40
32
24
16
8
Chemical Shift (ppm)

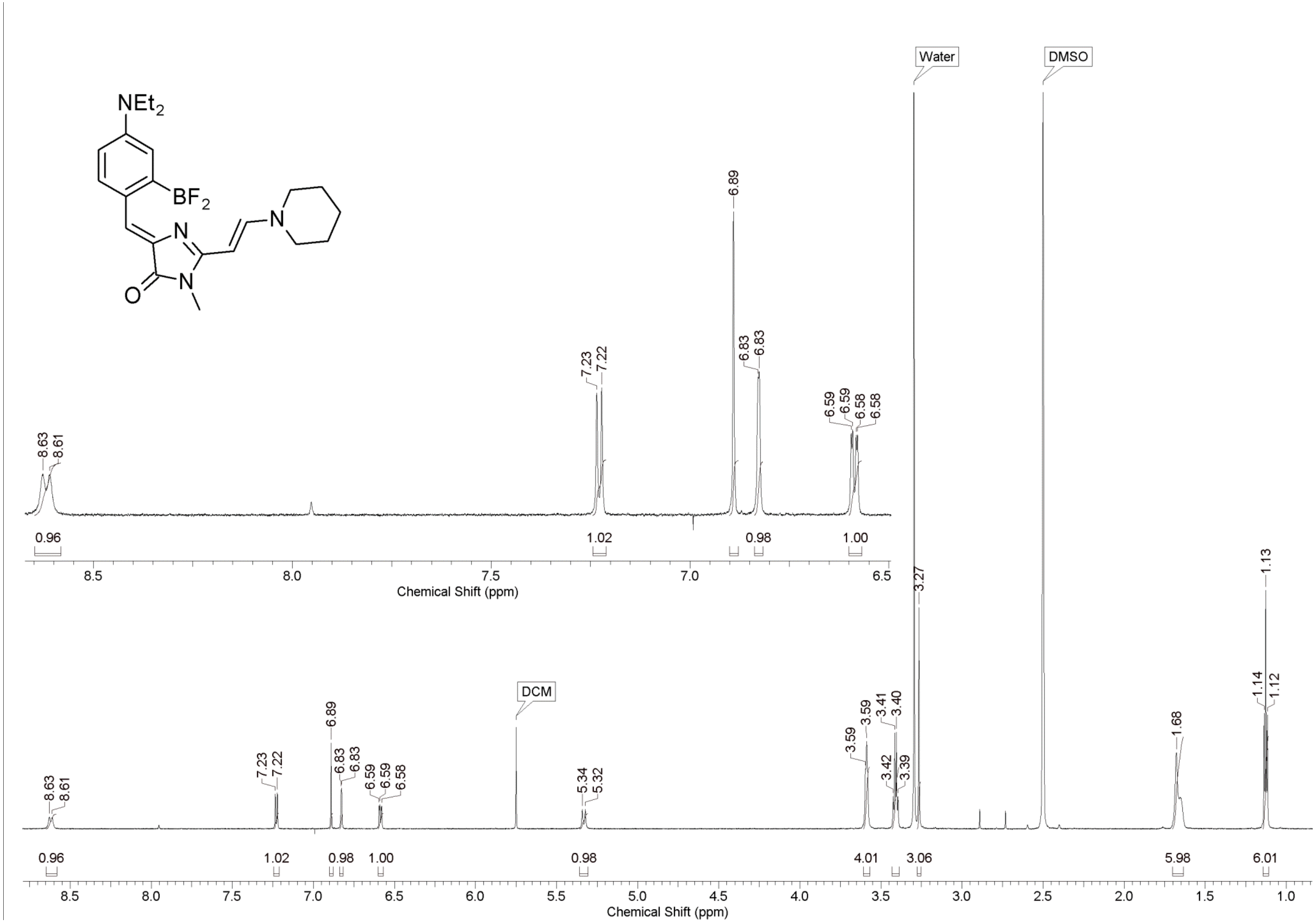
NEt2
BF2
N
N
N
O
Water
DMSO
DCM
8.63
8.61
7.23
7.22
6.89
6.83
6.83
6.59
6.59
6.58
6.58
5.34
5.32
3.59
3.59
3.41
3.40
3.42
3.39
3.27
1.68
1.14
1.13
1.12
0.96
1.02
0.98
1.00
4.01
3.06
5.98
6.01
Chemical Shift (ppm)
Chemical Shift (ppm)

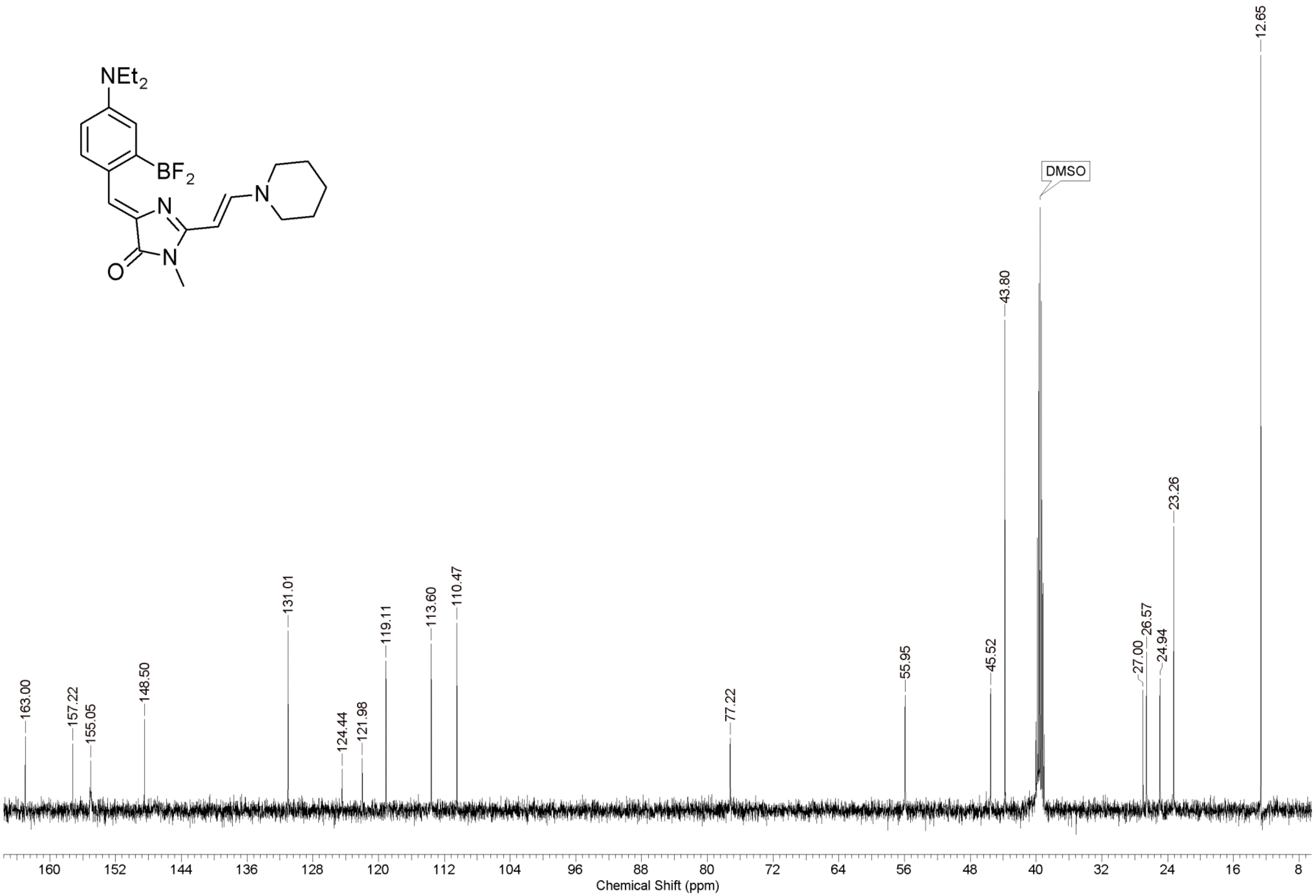
NEt2
BF2
N
N
N
O
DMSO
163.00
157.22
155.05
148.50
131.01
124.44
121.98
119.11
113.60
110.47
77.22
55.95
45.52
43.80
27.00
26.57
24.94
23.26
12.65
160 152 144 136 128 120 112 104 96 88 80 72 64 56 48 40 32 24 16 8
Chemical Shift (ppm)

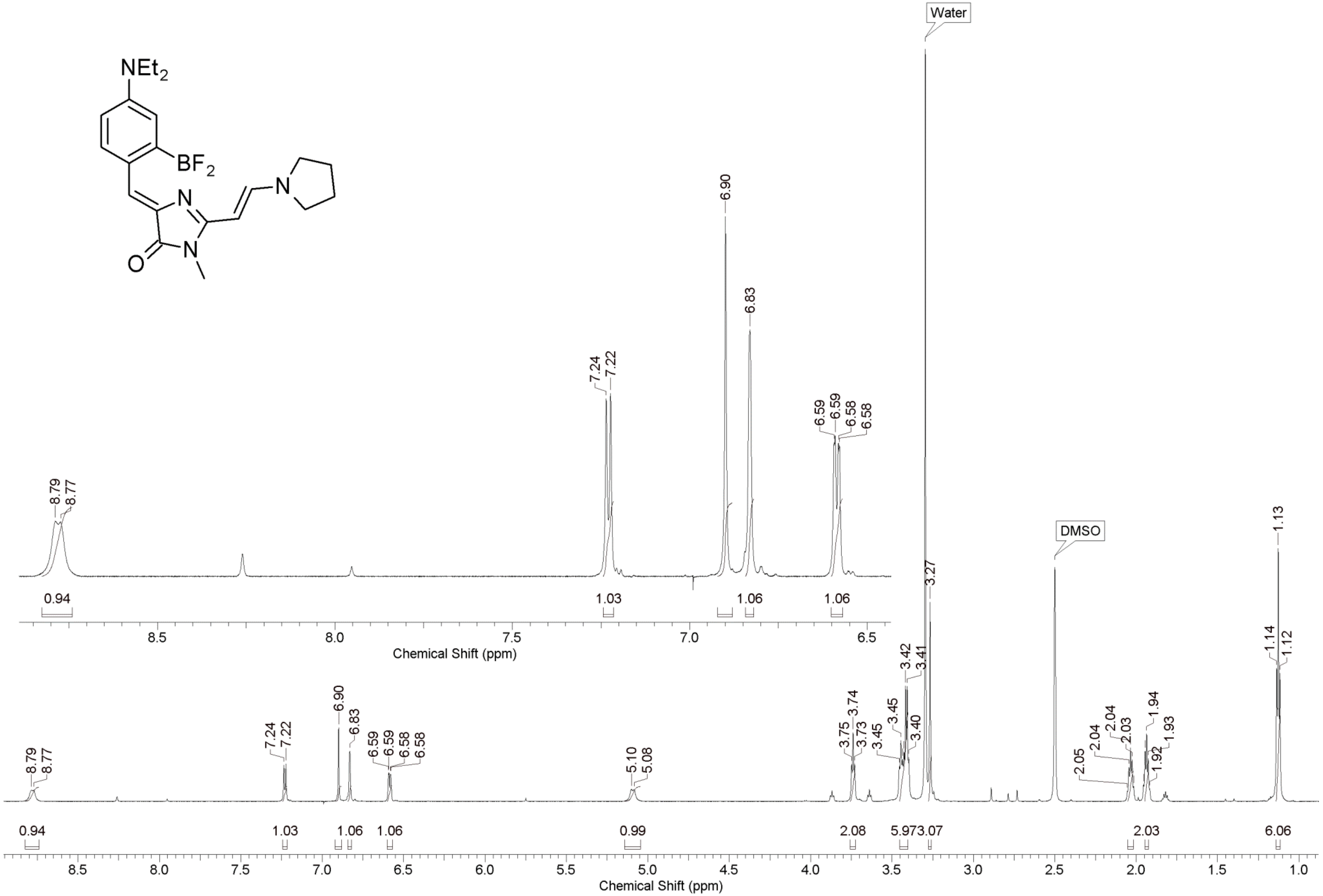

NEt2
BF2
N
O
Water
DMSO
Chemical Shift (ppm)
Chemical Shift (ppm)

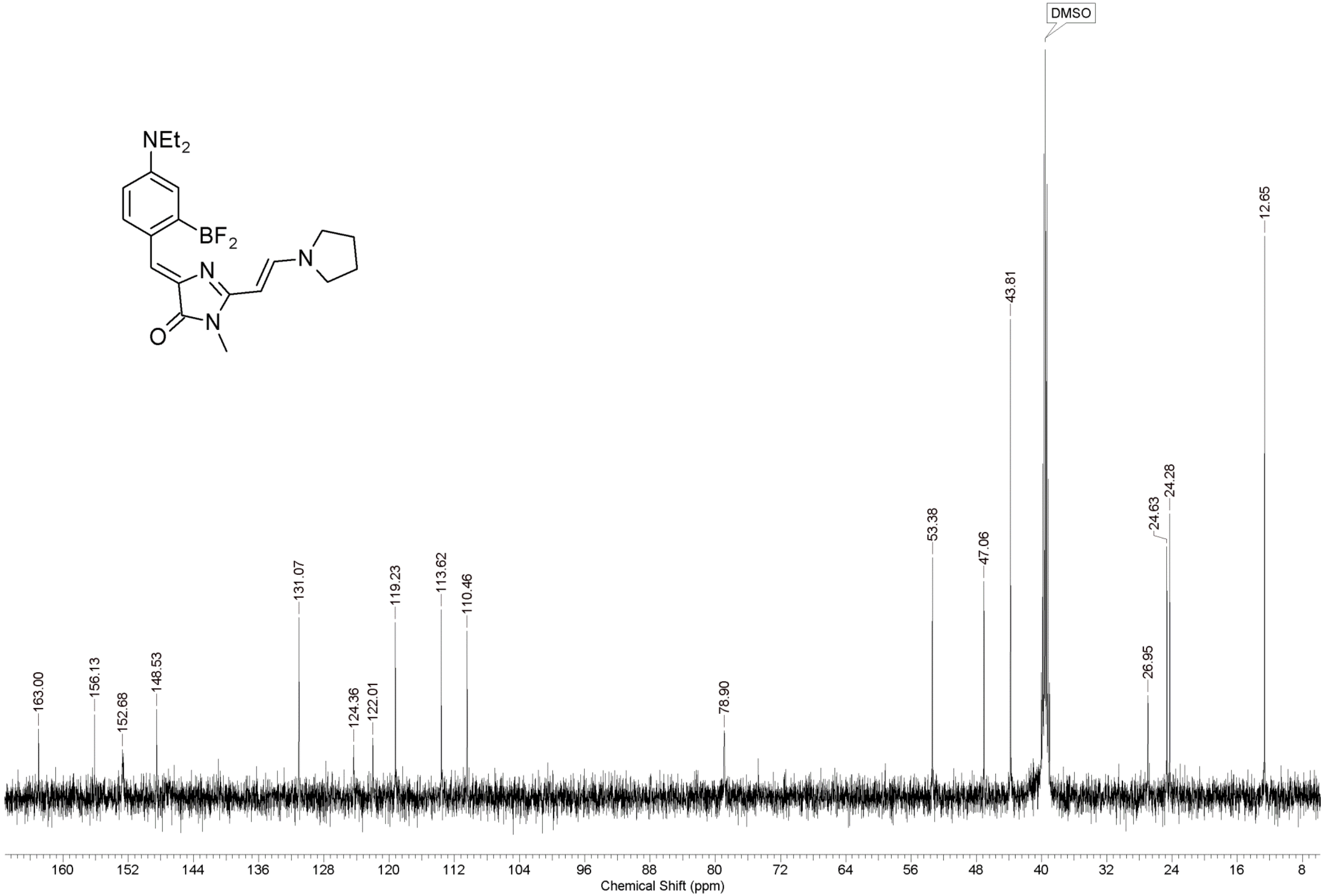
NEt2
BF2
N
N
O
DMSO
163.00
156.13
152.68
148.53
131.07
124.36
122.01
119.23
113.62
110.46
78.90
53.38
47.06
43.81
26.95
24.63
24.28
12.65
160
152
144
136
128
120
112
104
96
88
80
72
64
56
48
40
32
24
16
8
Chemical Shift (ppm)

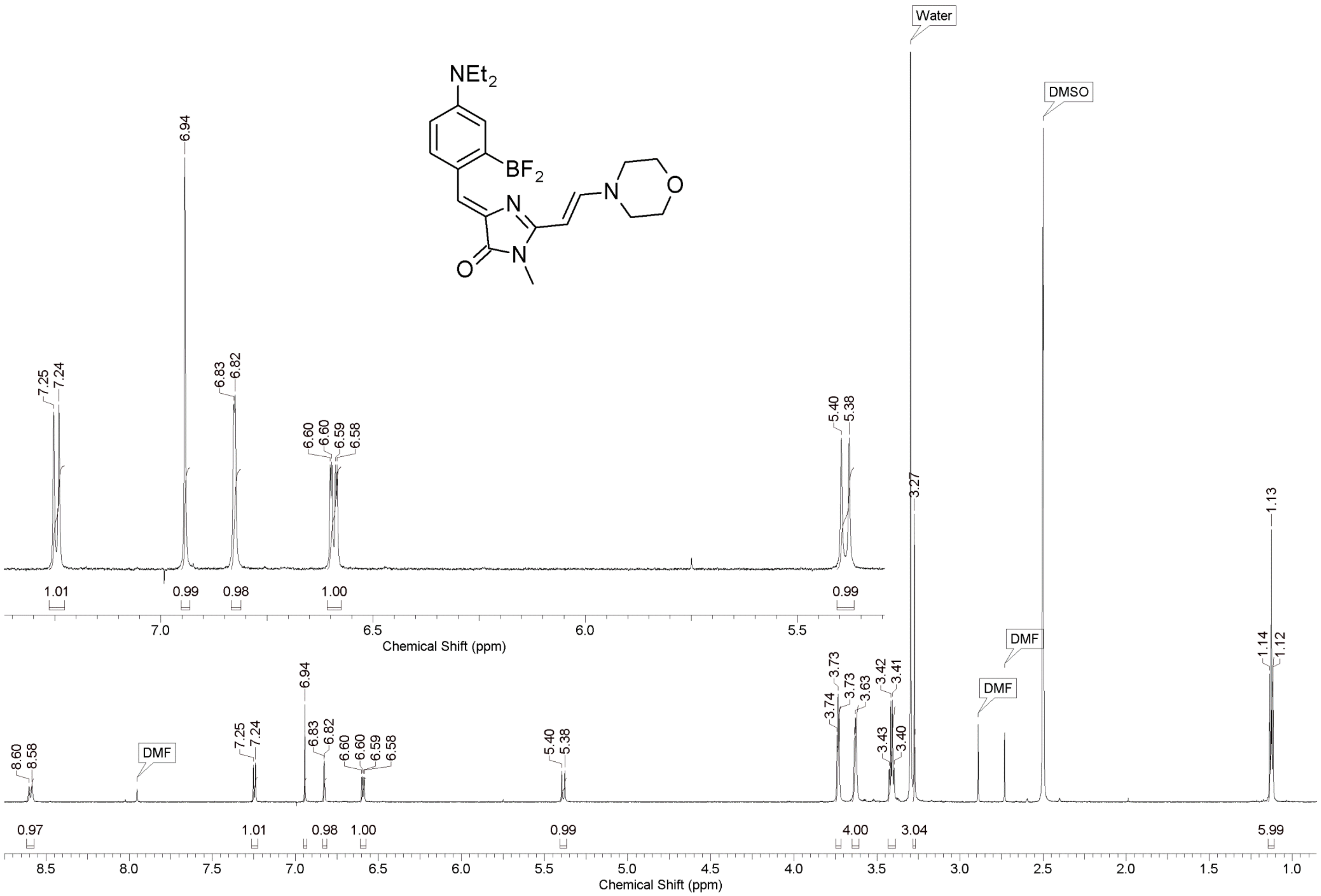

NEt2
BF2
N
N
O
N
O
Water
DMSO
DMF
DMF
DMF
Chemical Shift (ppm)
Chemical Shift (ppm)

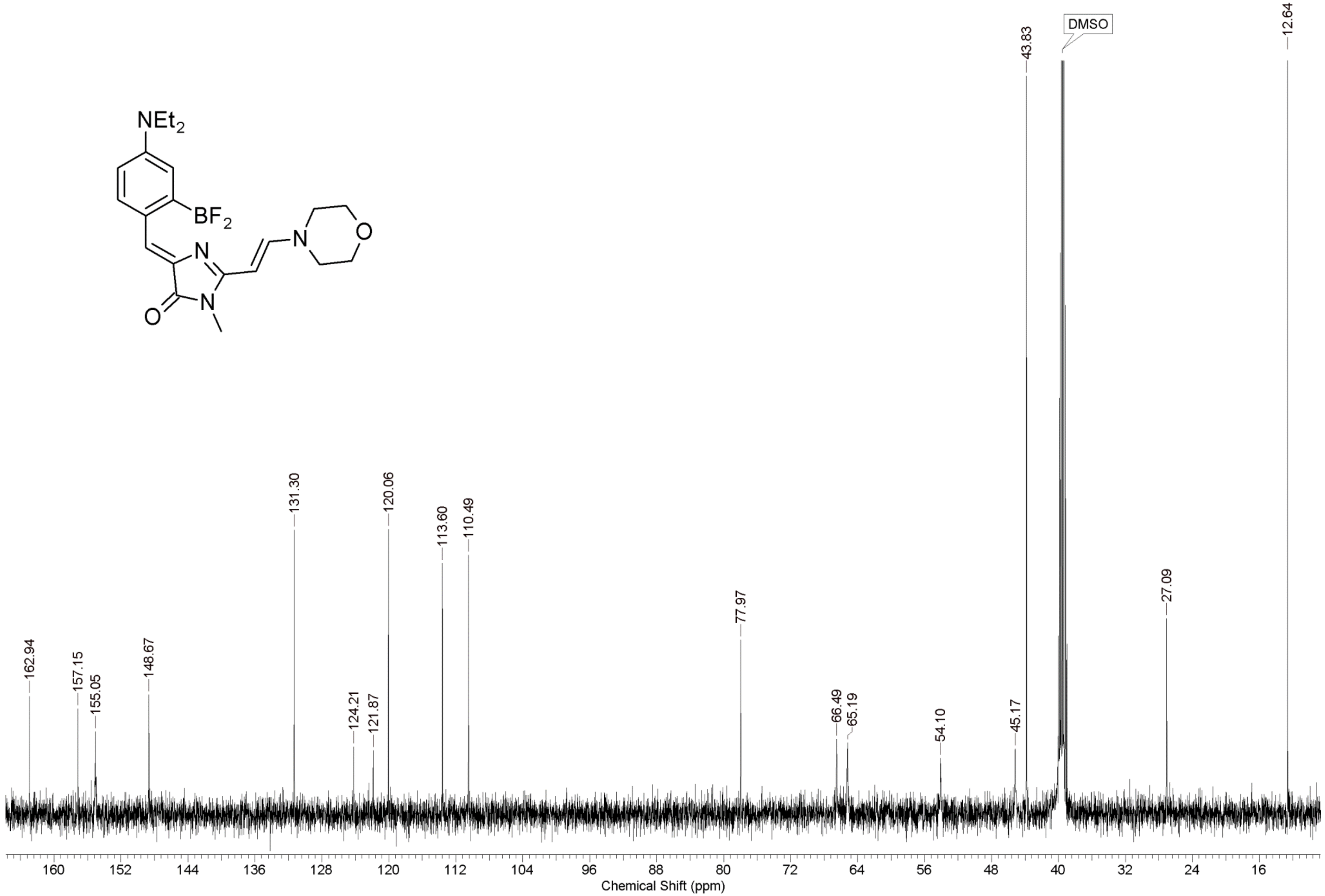
NEt2
BF2
N
N
O
N
O
DMSO
162.94
157.15
155.05
148.67
131.30
124.21
121.87
120.06
113.60
110.49
77.97
66.49
65.19
54.10
45.17
43.83
27.09
12.64
160 152 144 136 128 120 112 104 96 88 80 72 64 56 48 40 32 24 16
Chemical Shift (ppm)

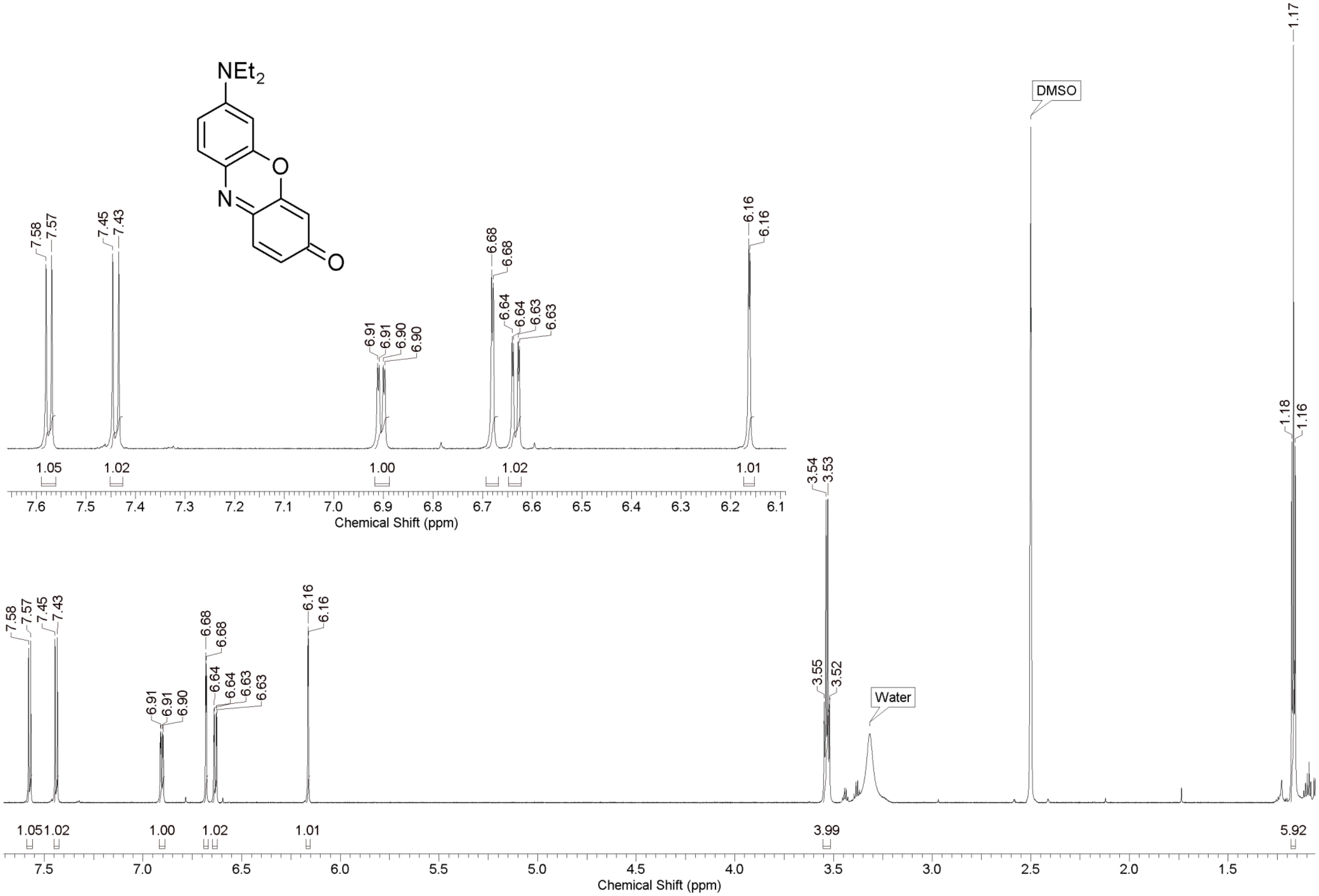
NEt2
O
N
O
DMSO
Water
1.17
1.18
1.16
3.54
3.53
3.55
3.52
7.58
7.57
7.45
7.43
6.91
6.91
6.90
6.90
6.68
6.68
6.64
6.64
6.63
6.63
6.16
6.16
1.05
1.02
1.00
1.02
1.01
3.99
5.92
Chemical Shift (ppm)
Chemical Shift (ppm)

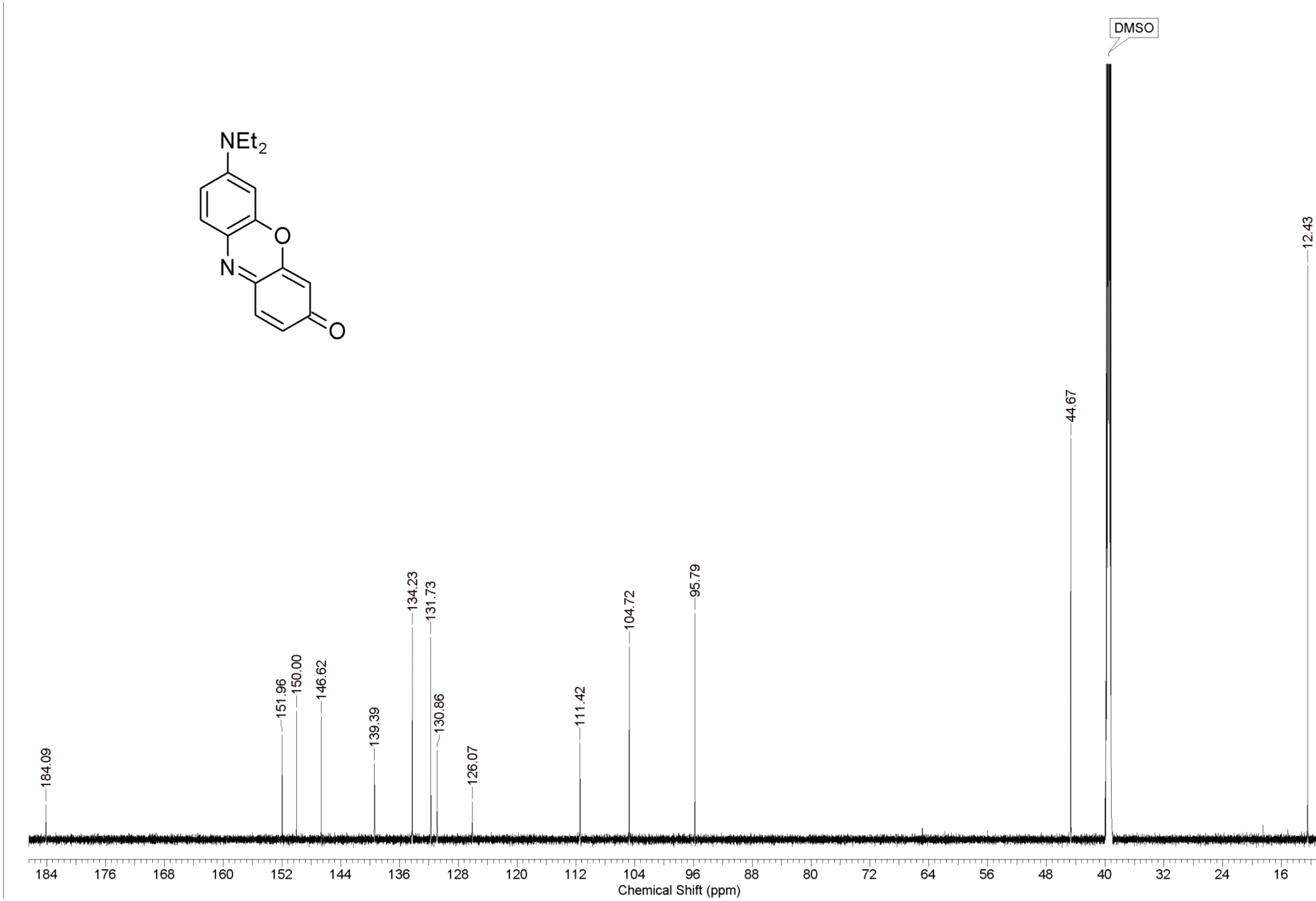
DMSO
NEt2
O
N
O
184.09
151.96
150.00
146.62
139.39
134.23
131.73
130.86
126.07
111.42
104.72
95.79
44.67
12.43
184 176 168 160 152 144 136 128 120 112 104 96 88 80 72 64 56 48 40 32 24 16
Chemical Shift (ppm)

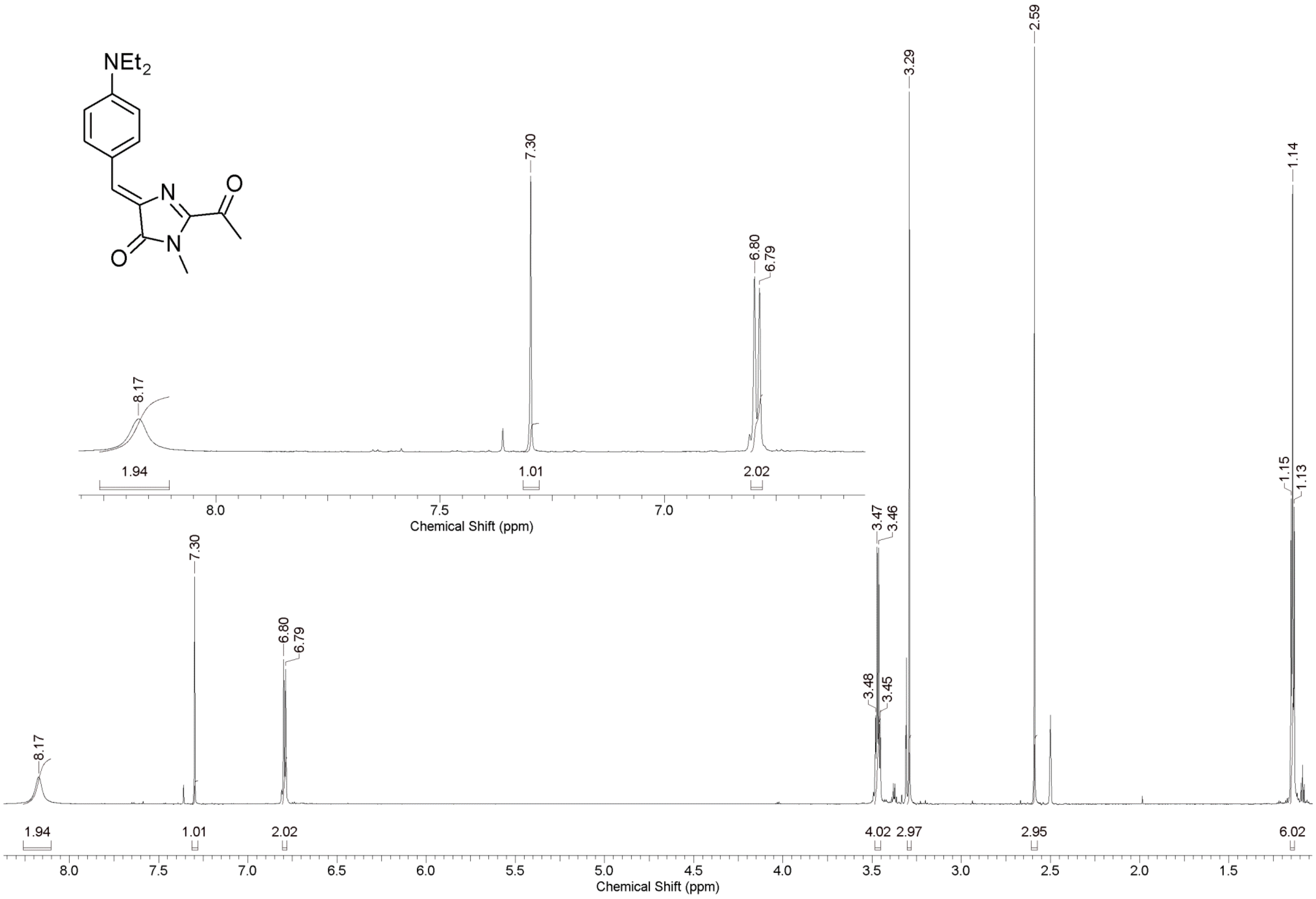
NEt2
Chemical Shift (ppm)
Chemical Shift (ppm)

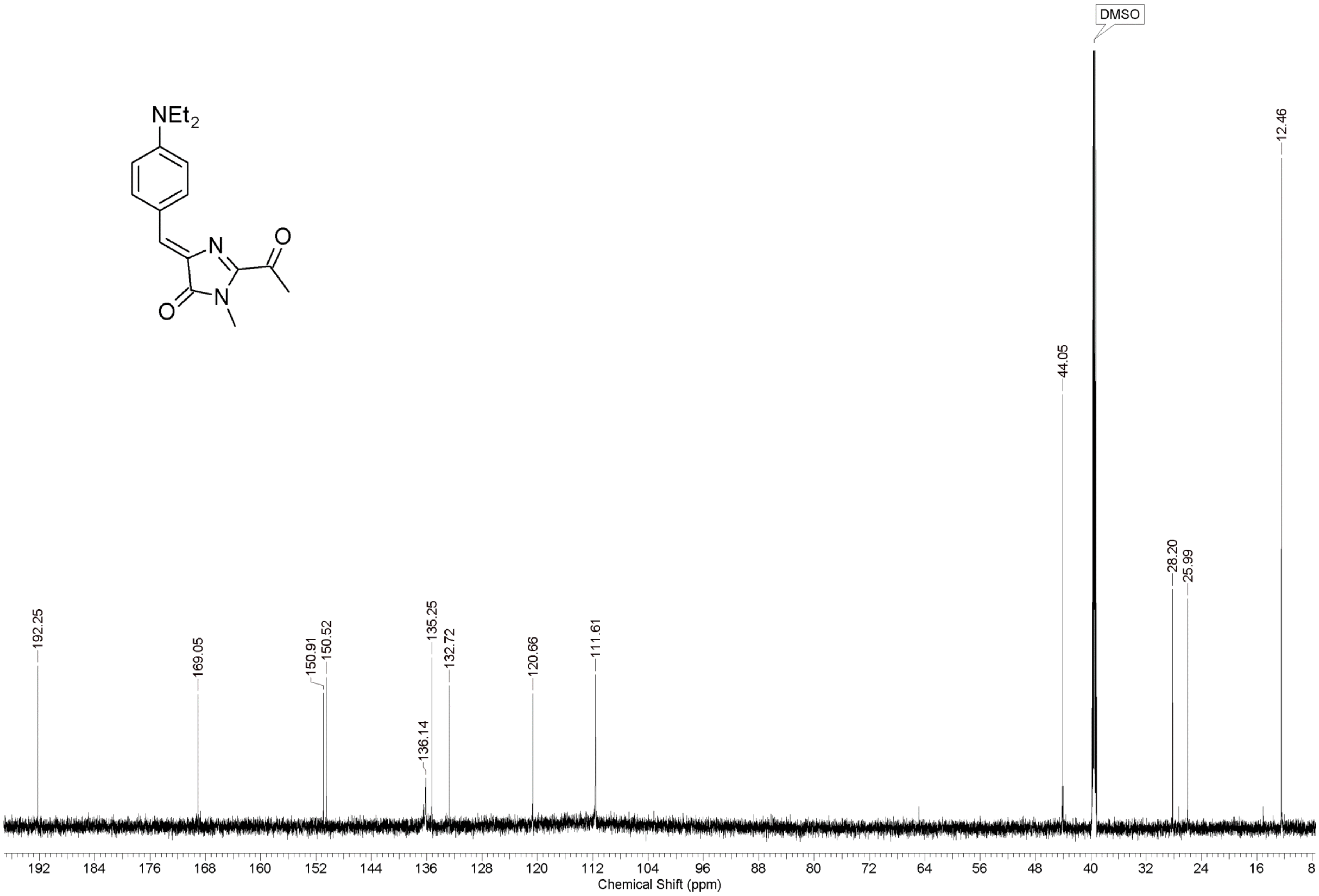
NEt2
N
O
N
O
DMSO
192.25
169.05
150.91
150.52
136.14
135.25
132.72
120.66
111.61
44.05
28.20
25.99
12.46
192 184 176 168 160 152 144 136 128 120 112 104 96 88 80 72 64 56 48 40 32 24 16 8
Chemical Shift (ppm)

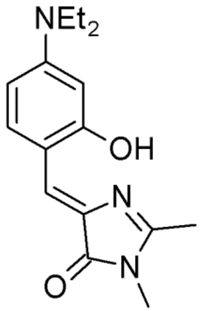
NEt2
OH
N
N
O

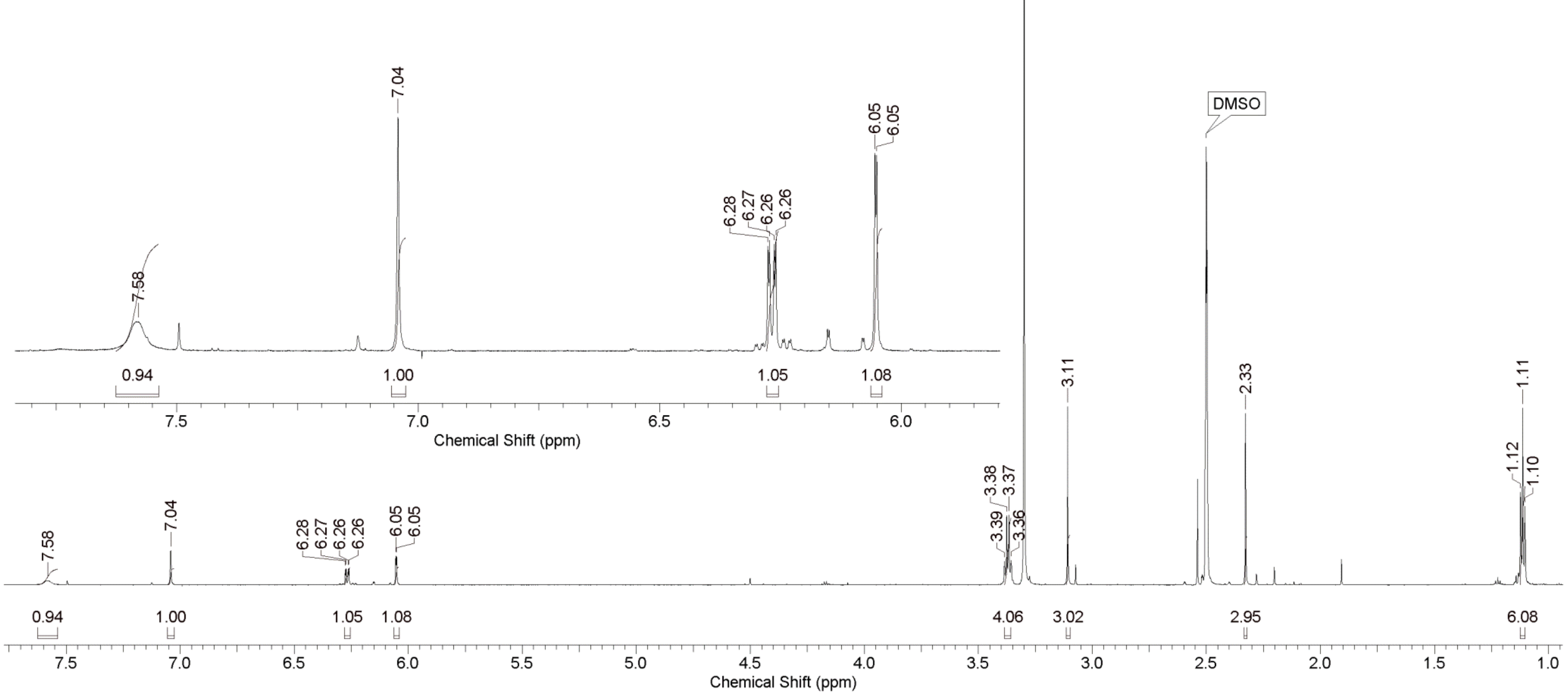
Water
DMSO
7.58
7.04
6.28
6.27
6.26
6.26
6.05
6.05
0.94
1.00
1.05
1.08
Chemical Shift (ppm)
3.39
3.38
3.37
3.36
3.11
2.33
1.12
1.11
1.10
4.06
3.02
2.95
6.08
Chemical Shift (ppm)

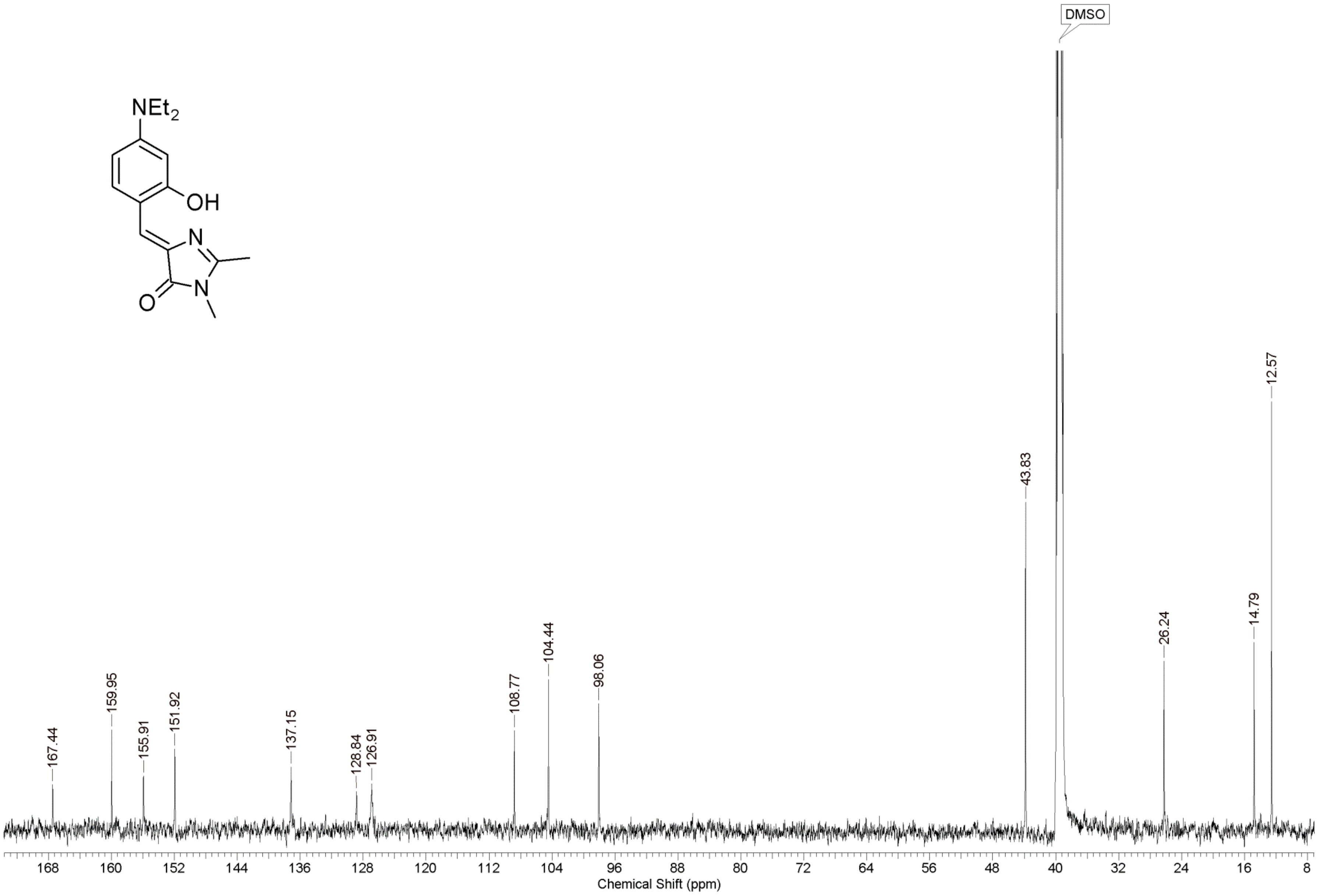
NEt2
OH
N
N
O
DMSO
167.44
159.95
155.91
151.92
137.15
128.84
126.91
108.77
104.44
98.06
43.83
26.24
14.79
12.57
168
160
152
144
136
128
120
112
104
96
88
80
72
64
56
48
40
32
24
16
8
Chemical Shift (ppm)
